# Health conditions that impact fitness-to-practice in physicians: a scoping review

**DOI:** 10.1093/intqhc/mzaf108

**Published:** 2025-10-07

**Authors:** Richard Roberts, Tanya Jackson, Ryan Gerdes, Danika Deibert, Liz Dennett, Ellina Lytvyak, Sebastian Straube

**Affiliations:** Division of Preventive Medicine, Department of Medicine, University of Alberta, Edmonton, AB, Canada; Division of Preventive Medicine, Department of Medicine, University of Alberta, Edmonton, AB, Canada; Division of Preventive Medicine, Department of Medicine, University of Alberta, Edmonton, AB, Canada; Division of Preventive Medicine, Department of Medicine, University of Alberta, Edmonton, AB, Canada; Geoffrey and Robyn Sperber Health Sciences Library, University of Alberta, Edmonton, AB, Canada; Division of Preventive Medicine, Department of Medicine, University of Alberta, Edmonton, AB, Canada; Division of Preventive Medicine, Department of Medicine, University of Alberta, Edmonton, AB, Canada; School of Public Health, University of Alberta, Edmonton, AB, Canada

**Keywords:** licensing of health professionals, patient safety, quality management

## Abstract

**Background:**

It has been over 50 years since health conditions in physicians were first suggested to affect their fitness-to-practice, with consequent impacts on patient safety and patient care. Recent policy positions from physician regulatory bodies express a desire for clarity regarding the impact of these health conditions alongside their standardization in physician regulatory processes. Furthermore, these conditions have not been fully enumerated. Therefore, this scoping review intended to find all health conditions which were identified in the literature to impact physician fitness-to-practice.

**Methods:**

A specialist librarian developed and executed a systematic literature search in Ovid MEDLINE, Embase via Ovid, APA PsycINFO, and ProQuest Dissertations & Theses Global (to January 2024). The SPIDER framework was used for inclusion criteria and records were screened independently by two reviewers by title and abstract, and then by full text. Any study addressing a health condition identified as able to affect fitness-to-practice in physicians and surgeons, physician assistants, or medical trainees was eligible.

**Results:**

In 403 eligible records of 2542 screened, 4336 total mentions of 203 fitness-to-practice-related health conditions were identified. Conditions relating to mental health issues (32.0%) and drug/substance use (26.0%) comprised more than half of the condition reports. This was followed by neurological conditions (13.2%), medical conditions (12.8%), alcohol use (6.0%), addiction (3.0%), and aging (2.9%) as well as conditions affecting dexterity/fine motor skills/psychomotor performance (2.1%), vision (1.4%), and hearing (0.6%).

**Conclusions:**

This scoping review identified a wide variety of health conditions which could affect physician fitness-to-practice, with a potential impact on patient care and safety. These conditions have persisted in the literature, and we commend them to the attention of practicing physicians, researchers, regulators, and physician health programs.

## Introduction

Throughout all stages of their careers, physicians are expected to maintain a standard of practice characterized by informed and patient-centered decision-making, which relies on their clinical knowledge and technical skill sets. As bearers of trust within a community, they are also expected to self-regulate in their assessment of these personal competencies—specifically, to understand when their capability to practice is reduced or diminished due to a health condition, and to correspondingly limit their professional activities. In addition to self-regulation, further aspects may include the involvement of colleagues, participation in physician health programs (PHPs), and disciplinary action from physician regulatory bodies. Whereas early landmark papers aiming to define physician fitness-to-practice referred specifically to those individuals exhibiting problematic substance use or psychological disorders [1], impairment as a concept has since expanded, and is more fully defined as “any physical, mental or behavioral disorder that interferes with the ability to engage safely in professional activities” [2]. Examples of this broader scope of physician impairment within the literature of the past 20 years include “cognitive function and serious mood disturbance” [3], “neuropsychological difficulty” [3], “aging” [3], “sleep, [and] burnout” [4], though these examples are evidently non-exhaustive. The expansion of this research field has coincided with policy positions from physician regulatory committees to provide clarity to the appropriateness of intervention due to health conditions, [5] as well as recommendations for better standardization of physician regulatory processes [6, 7]. This research need is important, especially given that physician impairment definitions exhibit substantial variation depending on jurisdiction [8], and physician health programs have materially expanded beyond their early scope of substance use disorder (SUD) treatment [9]. More recent findings regarding entrance into physician health programs showcase that SUD represents a minority (14.0%) of presenting problems, indicative of the need of a multifaceted and nuanced approach to management [10].

All of this underscores the concept that healthcare professionals—including physicians—are not immune to having health conditions of their own, and that such conditions may impact occupational performance, concerning their fitness-to-practice and carry-over effects on patient care. Determining the current scope of physician fitness-to-practice literature, with respect to all health conditions identified as potentially impacting fitness-to-practice, can serve medical licensure boards, physician regulatory agencies, and PHPs who wish to clarify their policies and procedures, especially given the increased focus on stricter, government-oriented health profession regulation in high-income countries [11]. In this context, we here report on a scoping review which we conducted about health conditions that may impact fitness-to-practice.

## Methods

This review followed Preferred Reporting Items for Systematic reviews and Meta-Analyses extension for Scoping Reviews (PRISMA-ScR) guidelines (see Supplementary File S1) [12].While our methodology was established *a priori*, an online review protocol was not published for this scoping review.

A systematic literature search was conducted by author Liz Dennett, a health sciences librarian, in Ovid MEDLINE (1946–16 January 2024), Embase via Ovid (1974–16 January 2024), APA PsycINFO (1806–Week 2 January 2024), and ProQuest Dissertations & Theses Global (via Web of Science; searched 17 January 2024). The literature search strategies are given in Supplementary Files S2–S5. The search strategy was developed and iteratively refined via targeted keyword searching within the chosen databases and was informed by the SPIDER inclusion criteria framework [13], as follows:

**S**ample: Physicians, surgeons, residents, fellows, physician assistants, and medical students
**P**henomenon of **I**nterest: Fitness-to-practice (impact on patient safety or patient care)
**D**esign: All study designs
**E**valuation: Identification of health conditions
**R**esearch type: Qualitative and quantitative, including conference abstracts, academic theses and dissertations, and peer-reviewed literature.

We furthermore restricted study inclusion to articles published 1 January 1994 or later, to ensure that the information retrieved was relevant to healthcare professionals currently in practice. We also constrained the language of articles’ full-text publications to English, French, and German.

We uploaded the records resulting from the literature search into Covidence, a web-based software for collaborative systematic and literature review management [14].

Following automatic record deduplication, record titles and abstracts were screened independently by two research team members to assess inclusion eligibility. Full texts of records deemed potentially eligible at the title/abstract stage were obtained. Screening at the full-text stage was then performed independently by two researchers, and reasons for exclusion were given for any records excluded at this stage. Disagreements at the title/abstract or full-text screening stages were resolved via consensus discussion, or arbitration by a senior research team member, Sebastian Straube, if necessary.

Each included record was assessed for all mentions of fitness-to-practice-related health conditions, with each newly identified health condition being appended as a new column of our data extraction spreadsheet according to the verbatim wording utilized in the record (row). Iteratively during the data extraction process, health conditions which utilized different verbatim wordings but were agreed upon by team members to indicate the same underlying diagnosis/pathology/phenomenon (e.g. “stroke” and “cerebrovascular accident”) were amalgamated together into a single individual condition. Health conditions were considered to have been mentioned on a per-record basis, in that multiple mentions of the same health condition within a single record were not recorded as separate mentions. This method resulted in the creation of a list of all fitness-to-practice-related health conditions identified within the included records, as well as counts of the number of records in which each health condition was mentioned.

Following data extraction, individual health conditions were grouped together in categories reflective of clinical practice. The aim of this grouping process was to synthesize the information while retaining sufficient detail to identify overarching themes and trends in the body of research. Lastly, the included articles were divided into two groups based on publication date, with the first group encompassing articles published from 1994 to 2018 (“non-recent articles”) and the second group articles published from 2019 onwards (“recent articles”). Chi-square tests with 95% confidence limits were used to assess absolute and relative differences in proportions for each condition category grouping between recent and non-recent articles.

## Results

**Figure 1:**
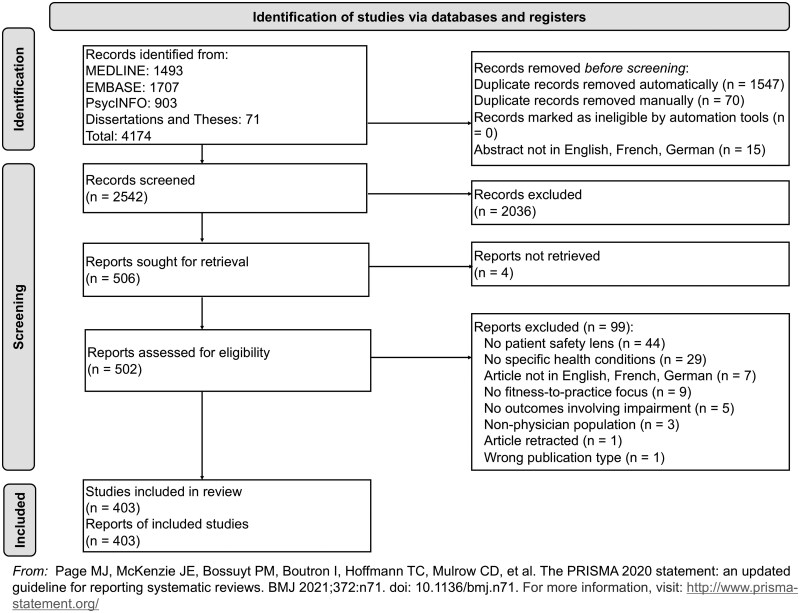
PRISMA flowchart for identification of articles which addressed physician fitness-to-practice-related health conditions

The study selection process is given in Fig. 1 [15]. A total of 4174 records were identified from the database search. Following deduplication, 2542 records were screened at the title and abstract stage. At this stage, 481 records with publication dates of 1993 or earlier were excluded.

In total, 506 studies remained after title and abstract screening and were sought for full-text retrieval, of which 502 were successfully obtained. At the full-text screening stage, 99 of these studies were excluded. The most common rationale for exclusion at the full-text stage was that the article did not provide any example of a health condition which was associated with a change to patient safety or patient care outcomes.

A total of 403 records were ultimately included and underwent data extraction (Supplementary File S6), whereupon 4336 total mentions of 203 fitness-to-practice-related health conditions were identified. This corresponded to a mean of 10.8 identified health conditions mentioned within each included record.

### Frequency of reported conditions

The most frequently reported condition (Table 1) was substance use, abuse, misuse, or addiction, and the ten most frequently reported conditions (Table 1) represented 39.1% of all condition reports. The 203 identified conditions were further classified into ten categories (Table 2). Mental health conditions (55 constituent conditions), alcohol use and related conditions (3 constituent conditions), and drug/substance use and related conditions (61 constituent conditions) closely reflect clinically similar diagnostic groupings. The categories of aging and age-related issues (2 constituent conditions) as well as conditions affecting dexterity/fine motor skills/psychomotor performance (1 constituent condition), vision (1 constituent condition), and hearing (1 constituent condition) were presented in a more verbatim fashion to ensure fidelity to the language of the included studies and to best represent the observed findings. The constituent conditions associated with each of the categories are presented in Supplementary File S7.

**Table 1.** Top 10 most frequently reported individual fitness-to-practice-related health conditions (different verbatim terms for the same phenomenon, diagnosis, or pathology were amalgamated), by frequency

Rank	Individual health condition	Percent of all reports (%); number of instances (*n*)
1	Substance use/substance abuse/substance misuse/substance addiction	5.6% (264)
2	Alcohol use/alcohol misuse/alcohol dependence/alcohol addiction/alcoholism/drinking problem	5.0% (224)
3	Mental health/mental illness/mental disorder/psychological health/emotional health	4.9% (217)
4 (tie)	Depression	3.7% (162)
	Cognitive change/decline	3.7% (162)
6 (tie)	Psychiatric disorders/psychiatric conditions/psychological disorders/psychological conditions	3.2% (144)
	Physical illness/physical health problem/physical condition/physical diagnosis/physical disease	3.2% (144)
8	Addiction (not further specified)	3.0% (132)
9	Aging	2.8% (126)
10	Stress	2.7% (121)

**Table 2.** Fitness-to-practice-related health condition categories, by reporting frequency

Fitness-to-practice condition category	Percent of all reports; number of instances (*n*)
Mental health issues	32.0% (1387)
Drug/substance use and related conditions	26.0% (1128)
Neurological conditions	13.2% (572)
Medical conditions (other than neurological, hearing, vision)	12.8% (554)
Alcohol use and related conditions	6.0% (259)
Addiction (not further specified)	3.0% (132)
Aging and age-related issues	2.9% (128)
Dexterity/fine motor skills/psychomotor performance	2.1% (90)
Vision	1.4% (60)
Hearing	0.6% (26)

### Temporal trends

The 403 included articles were divided into two groups based on publication date. This resulted in 3398 non-recent mentions and 938 recent mentions.

The relative proportions (contributions) of most categories did not differ from 1994 to 2018 versus 2019 onwards (Table 3), with the exception of neurological conditions, where the relative frequency of mentions was higher among recent articles.

**Table 3.** Fitness-to-practice-related health condition categories, by proportion of total condition mentions, 1994–2018 versus 2019–onwards

Condition category	Proportion (*n*) of total mentions within non-recent articles (published 1994–2018) *N* = 3398	Proportion (*n*) of total mentions within recent articles (published 2019 onwards) *N* = 938	Absolute proportion difference (point estimate, 95% confidence interval)	Relative prevalence ratio (Recent versus non-recent) (point estimate, 95% confidence interval)
Addiction (not further specified)	3.2% (109)	2.5% (23)	−0.7% (−1.9% to 0.3%)	0.76 (0.49 to 1.19)
Aging and age-related issues	2.9% (97)	3.3% (31)	0.4% (−0.8% to 1.7%)	1.15 (0.78 to 1.72)
Dexterity/fine motor skills/psychomotor performance	1.9% (63)	2.9% (27)	1.0% (−0.1% to 2.2%)	1.55 (0.99 to 2.42)
Hearing	0.64% (20)	0.59% (6)	0.05% (−0.5% to 0.6%)	1.09 (0.43 to 2.70)
Vision	1.4% (46)	1.5% (14)	0.1% (−0.7% to 1.0%)	1.10 (0.61 to 2.00)
Alcohol use and related conditions	6.3% (213)	4.9% (46)	1.4% (−3.0% to 0.2%)	0.78 (0.57 to 1.07)
Drug/substance use and related conditions	26.2% (890)	25.4% (238)	−0.8% (−4.0% to 2.3%)	0.97 (0.86 to 1.10)
Mental health issues	32.1% (1092)	31.4% (295)	−0.6% (−4.0% to 2.7%)	0.98 (0.88 to 1.09)
Neurological conditions	12.4% (420)	16.2% (152)	**3.8% (1.2% to 6.4%)**	**1.31 (1.11 to 1.56)**
Medical conditions (other than neurological, hearing, vision)	13.2% (448)	11.3% (106)	−1.9% (−4.2% to 0.4%)	0.86 (0.70 to 1.05)
Bolding indicates statistical significance (*P* < .05).				

## Discussion

### Statement of principal findings

To our knowledge, this is the first scoping review to comprehensively assess the physician fitness-to-practice literature, recording the mentions of any health condition potentially impacting patient safety or care. We found that the majority of references to fitness-to-practice-related concerns corresponded to one of the three prototypical health condition categories—psychiatric illness, alcoholism, and drug use—with their constituent category groupings making up 64.0% of all health condition mentions [1]. Whether these three broad categories remain a focus of the research community due to their relatively common prevalence among the physician community, the effect of early discourse on the topic, or a combination of both remains to be assessed [1, 16, 17].

### Interpretation within the context of the wider literature

We documented mental health concerns as the most common health condition category within the physician fitness-to-practice literature. This finding is congruent with the focus of recent reviews which have sought to assess physician mental health support systems [18–20], alongside individual-level literature seeking to identify workplace support components—including approachable leadership and perceived peer support—predictive of physician mental health in a post-pandemic setting [21]. Notably, Kolobaric and colleagues identified improvements among physician mental health domains following “three quarters of the educational interventions, all of the physical interventions, half of the administrative interventions, two-thirds of the social interventions and just over half of multifactorial interventions” [18]. Furthermore, Carreiri and colleagues concluded that “functional” physician working groups can reduce mental ill-health [19], and work by Petrie and colleagues supplements these conclusions, identifying “group-based, face to face and skills-based interventions” as potential avenues to manage symptoms of common mental disorders among physicians [20].

The relative proportion of each health condition category grouping in our review can be seen as a reflection of trends in contemporary fitness-to-practice research. This is notable, as discourse regarding these conditions—or condition categories—may be subject to shifts in focus as their management changes through increased access to newer and more effective treatment modalities. Our analysis sought to identify any such changes, by assessing differences in the proportion of mentions pertaining to health condition categories between older (1994–2018) and more recent (2019 onwards) articles. We found that statistically significant changes in proportion of mention only took place for neurological conditions, and we conclude that the physician fitness-to-practice literature showcases relatively strong temporal stability.

### Implications for policy, practice, and research

This scoping review is best viewed as presenting health conditions which the literature suggests may contribute to impaired fitness-to-practice among physicians and is in accord with prior position statements which acknowledge gaps in fitness-to-practice definitions and a need for further clarification [5, 9]. In contrast, this review should not be misconstrued to have identified health conditions which necessitate disclosure to a regulatory body, enrollment in monitoring programs, or engagement in disciplinary action, given that we do not describe potentially mediating factors such as condition severity, disease course, or available workplace programs. Moreover, differences in physician specialization or practice scope may also affect which conditions are impactful. Given the potential for variability of impact from the same condition by name, a tiered response to fitness-to-practice concerns—proportional to any possible or realized risk to patients—remains a recommended approach by multiple physician regulatory bodies [5, 7]. Such a tiered response may specifically promote the initiation of help-seeking for some psychiatric conditions versus more stringent health reporting requirements, which has been shown to reduce these behaviors [22, 23].

### Strengths and limitations

This study benefits from a thoughtfully designed methodology which we specifically tailored to promote the inclusion of relevant articles within our scoping review. We opted to use the SPIDER inclusion criteria framework [13] to most accurately contextualize our topic of interest, and we iteratively developed our search strategy using an extensive list of fitness-to-practice-related keywords and concepts in order to maximize our search exposure with respect to the aggregate body of physician fitness-to-practice literature. Furthermore, we chose to summarize the most frequently mentioned health conditions at two levels of granularity—single condition and condition category—to provide clarity with respect to their linkage, to facilitate their comparison with one another, and to allow decision-makers to utilize the most appropriate type of data in future research and policy.

There are limitations to the evidence that was included in this review, two of which we believe warrant further discussion: choice of article publication date, and use of language restrictions. Our choice to assess the body of fitness-to-practice-related literature specifically via articles published in 1994 and onwards prohibits the assessment of longer-term temporal changes in research focus (e.g. 1970s through the early 1990s). However, this represents a deliberate choice, in that we believe these newer articles are most representative of the practices of currently working physicians, and that the research body encompassing 1994 and onwards likely captures all currently relevant fitness-to-practice-related concerns to an acceptably sufficient degree. The present scoping review also restricted included articles to those published in English, French, or German, and thus may not fully generalize to countries with other languages of research publication. However, our choice to limit by language is supported by meta-epidemiological research which indicates that rapid and systematic reviews are unlikely to generate different conclusions via the inclusion or exclusion of non-English articles [24]. In addition, while our review faithfully captured verbatim wording used to describe health conditions of significance to patient care, we did not seek to capture how these health conditions could be impacted by individual or workplace-level factors identified by the authors of the studies examined by us. Such factors may or may not be modifiable, and could have included educational, physical, administrative, or social factors at the workplace level [18], and comorbidity, demographic, and personality factors at the individual level. Exploring this further remains a task for future research.

## Conclusion

This scoping review set out to identify health conditions that have been viewed as impacting physician fitness-to-practice, specifically focusing on the outcomes of negative effects on patient care and patient safety. Our literature search found 203 health conditions which were identified as having potential fitness-to-practice impacts. Given the sustained discourse regarding these health conditions, we suggest that those mentioned most frequently, whether on an individual basis or by clinical practice category, are worthy of consideration by clinicians, researchers, and regulators.

## Supplementary Material

mzaf108_Supplementary_Data

## Data Availability

The data underlying this work is derived from previously published sources (see Online Supplementary Appendix) and is therefore available to readers.
