# Supplementary material for: Health conditions that impact fitness-to-practice in physicians: a scoping review
Source: Int J Qual Health Care. 2025 Oct 7;37(4):mzaf108. doi: 10.1093/intqhc/mzaf108 (PMC12596707; doi:10.1093/intqhc/mzaf108)
Supplement: mzaf108_Supplementary_Data [file mzaf108_supplementary_data.zip › Supplemental File 4.docx]

# Supplemental File 4

## Embase

**1974 to 2024 January 16> (searched Jan 17, 2024)**

1 exp *physician/ 208013

2 *medical school/ or *resident/ or residency education/ 67310

3 *medical student/ 36564

4 ("medical resident*" or "medical student*" or "medical graduate*" or "post graduate trainee*" or physician* or "general practitioner*" or GP or doctor* or surgeon* or clinician* or internist* or specialist* or anaesthetist* or anesthesiologist* or cardiologist* or dermatologist* or diabetologist* or endocrinologist* or gastroenterologist* or geriatrician* or gerontologist* or gynaecologist* or gynecologist* or haematologist* or hematologist* or hepatologist* or immunologist* or neonatologist* or nephrologist* or neurologist* or neurosurgeon* or obstetrician* or ophthalmologist* or oncologist* or orthopedist* or otolaryngologist* or paediatrician* or pediatrician* or pathologist* or podiatrist* or physiatrist* or psychiatrist* or pulmonologist* or radiologist* or rheumatologist* or urologist* or ((resident or residents) adj2 (anesthesiology or cardiology or dermatology or endocrinology or gastroenterology or gerontology or gynaecology or gynecology or haematology or hematology or hepatology or immunology or neonatology or nephrology or neurology or neurosurgery or obstetrics or ophthalmology or oncology or orthopedic* or orthopaedic* or otolaryngology or paediatric* or pediatric* or pathology or podiatry or physiatry or psychiatry or pulmonology or radiology or rheumatology or urology))).ti,kf. 422019

5 1 or 2 or 3 or 4 568696

6 5 and ((physician* or "general practitioner*" or GP or doctor* or surgeon* or clinician* or internist* or specialist* or anaesthetist* or anesthesiologist* or cardiologist* or dermatologist* or diabetologist* or endocrinologist* or gastroenterologist* or geriatrician* or gerontologist* or gynaecologist* or gynecologist* or haematologist* or hematologist* or hepatologist* or immunologist* or neonatologist* or nephrologist* or neurologist* or neurosurgeon* or obstetrician* or ophthalmologist* or oncologist* or orthopedist* or otolaryngologist* or paediatrician* or pediatrician* or pathologist* or podiatrist* or physiatrist* or psychiatrist* or pulmonologist* or radiologist* or rheumatologist* or urologist*) adj4 (impaired or impairment*)).ti,kf. 567

7 5 and (("medical resident*" or "medical student*" or "medical graduate*" or "post graduate trainee*" or anesthesiology resident* or cardiology resident* or dermatology resident* or endocrinology resident* or gastroenterology resident* or gerontology resident* or gynaecology resident* or gynecology resident* or haematology resident* or hematology resident* or hepatology resident* or immunology resident* or neonatology resident* or nephrology resident* or neurology resident* or neurosurgery resident* or obstetrics resident* or ophthalmology resident* or oncology resident* or orthopedic* resident* or orthopaedic* resident* or otolaryngology resident* or paediatric* resident* or pediatric* resident* or pathology resident* or podiatry resident* or physiatry resident* or psychiatry resident* or pulmonology resident* or radiology resident* or rheumatology resident* or urology resident*) adj4 (impaired or impairment*)).ti,kf. 16

8 5 and ((physician* or "general practitioner*" or GP or doctor* or surgeon* or clinician* or internist* or specialist* or anaesthetist* or anesthesiologist* or cardiologist* or dermatologist* or diabetologist* or endocrinologist* or gastroenterologist* or geriatrician* or gerontologist* or gynaecologist* or gynecologist* or haematologist* or hematologist* or hepatologist* or immunologist* or neonatologist* or nephrologist* or neurologist* or neurosurgeon* or obstetrician* or ophthalmologist* or oncologist* or orthopedist* or otolaryngologist* or paediatrician* or pediatrician* or pathologist* or podiatrist* or physiatrist* or psychiatrist* or pulmonologist* or radiologist* or rheumatologist* or urologist*) adj3 (impaired or impairment*)).ab. 369

9 5 and (("medical resident*" or "medical student*" or "medical graduate*" or "post graduate trainee*" or anesthesiology resident* or cardiology resident* or dermatology resident* or endocrinology resident* or gastroenterology resident* or gerontology resident* or gynaecology resident* or gynecology resident* or haematology resident* or hematology resident* or hepatology resident* or immunology resident* or neonatology resident* or nephrology resident* or neurology resident* or neurosurgery resident* or obstetrics resident* or ophthalmology resident* or oncology resident* or orthopedic* resident* or orthopaedic* resident* or otolaryngology resident* or paediatric* resident* or pediatric* resident* or pathology resident* or podiatry resident* or physiatry resident* or psychiatry resident* or pulmonology resident* or radiology resident* or rheumatology resident* or urology resident*) adj4 (impaired or impairment*)).ab. 29

10 5 and ("fit for practice" or "fit for practise" or "unfit for practice" or "unfit for practise" or "fit to practice" or "fit to practise" or "unfit to practice" or "unfit to practise" or "fitness to practice" or "fitness to practise" or "fitness for practice" or "fitness for practise" or "fit for duty" or "unfit for duty" or "fitness for duty").mp. 192

11 5 and "physician health program*".mp. 96

12 5 and (disclos* adj5 (recertification or certification or licensing authorit* or relicens* or revalidat* or medical licens* or "health condition*" or "medical condition*" or "physical health" or "mental health" or "physical illness*" or "mental illness*" or "psychiatric illness*" or disab* or impairment* or impaired or suicid* or "substance abuse" or "drug abuse" or "alcohol abuse" or "substance use" or abusing drug* or "recreational drug use*" or "illegal drug use*" or alcoholism or alcoholic* or burnout)).mp. 103

13 5 and ((age or aging or older or late-career) adj3 (physician* or "general practitioner*" or GP or doctor* or surgeon* or clinician* or internist* or specialist* or anaesthetist* or anesthesiologist* or cardiologist* or dermatologist* or diabetologist* or endocrinologist* or gastroenterologist* or geriatrician* or gerontologist* or gynaecologist* or gynecologist* or haematologist* or hematologist* or hepatologist* or immunologist* or neonatologist* or nephrologist* or neurologist* or neurosurgeon* or obstetrician* or ophthalmologist* or oncologist* or orthopedist* or otolaryngologist* or paediatrician* or pediatrician* or pathologist* or podiatrist* or physiatrist* or psychiatrist* or pulmonologist* or radiologist* or rheumatologist* or urologist*)).ti,kf. and (relicens* or re-licens* or recertif* or re-certif* or "return to practice" or remediation or reintegration or medical licens* or sanctions or competen* or incompetence or unprofessional* or professionalism or professional behavio* or impaired or impairment* or licensing authorit* or board certification or board citation* or erasure or suspension).mp. 146

14 5 and ((remediation or reintegration or relicens* or re-licens* or recertif* or re-certif* or "return to practice" or medical licens* or sanctions or suspension or suspensions or erasure or "disciplinary action*" or "regulatory authorit*" or "regulatory bod*" or "licensing authorit*" or "professional competence" or "physician competence" or incompetence or dyscompeten* or unprofessional* or professionalism or professional behavio*).ti,kf. and (((aging or older or late-career or well-being or health or cognitive ability or cognitive decline) adj3 (physician* or "general practitioner*" or GP or doctor* or surgeon* or clinician* or internist* or specialist* or anaesthetist* or anesthesiologist* or cardiologist* or dermatologist* or diabetologist* or endocrinologist* or gastroenterologist* or geriatrician* or gerontologist* or gynaecologist* or gynecologist* or haematologist* or hematologist* or hepatologist* or immunologist* or neonatologist* or nephrologist* or neurologist* or neurosurgeon* or obstetrician* or ophthalmologist* or oncologist* or orthopedist* or otolaryngologist* or paediatrician* or pediatrician* or pathologist* or podiatrist* or physiatrist* or psychiatrist* or pulmonologist* or radiologist* or rheumatologist* or "urologist* medical resident*" or "medical student*" or "medical graduate*" or "post graduate trainee*" or anesthesiology resident* or cardiology resident* or dermatology resident* or endocrinology resident* or gastroenterology resident* or gerontology resident* or gynaecology resident* or gynecology resident* or haematology resident* or hematology resident* or hepatology resident* or immunology resident* or neonatology resident* or nephrology resident* or neurology resident* or neurosurgery resident* or obstetrics resident* or ophthalmology resident* or oncology resident* or orthopedic* resident* or orthopaedic* resident* or otolaryngology resident* or paediatric* resident* or pediatric* resident* or pathology resident* or podiatry resident* or physiatry resident* or psychiatry resident* or pulmonology resident* or radiology resident* or rheumatology resident* or urology resident*)) or "health condition*" or "medical condition*" or "physical health" or "mental health" or illness* or depression or depressed or disab* or impairment* or impaired or suicid* or "substance abuse" or "drug abuse" or "alcohol abuse" or "substance use" or abusing drug* or "recreational drug use*" or "illegal drug use*" or alcoholism or alcoholic* or burnout).mp.) 397

15 6 or 7 or 8 or 9 or 10 or 11 or 12 or 13 or 14 1707
