# Supplementary material for: Health conditions that impact fitness-to-practice in physicians: a scoping review
Source: Int J Qual Health Care. 2025 Oct 7;37(4):mzaf108. doi: 10.1093/intqhc/mzaf108 (PMC12596707; doi:10.1093/intqhc/mzaf108)
Supplement: mzaf108_Supplementary_Data [file mzaf108_supplementary_data.zip › Supplemental File 6.docx]

1. Ackerman TF. Chemically dependent physicians and informed consent disclosure. *Journal of addictive diseases*. 1996;15(2):25-42.

2. Ackerman TF, Wall HP. A programme for treating chemically dependent medical students. *Medical education*. 1994;28(1):40-7.

3. Adhiyaman V, Hobson P, Sundaram R, Williams L. What are the precise reasons for the disparity in referrals to fitness to practise between international and UK medical graduates? *The Medico-legal journal*. 2023;91(4):198-203. doi:https://dx.doi.org/10.1177/00258172231184551

4. Adler RG, Constantinou C. Knowing - or not knowing - when to stop: cognitive decline in ageing doctors. *The Medical journal of Australia*. 2008;189(11-12):622-4.

5. Aiken LH, Dahlerbruch JH. Physician age and patient outcomes. *BMJ (Online)*. 2017;357((Aiken, Dahlerbruch) Center for Health Outcomes and Policy Research, School of Nursing, University of Pennsylvania, 418 Curie Boulevard, Philadelphia, PA 19104-4217, United States):j2286. doi:https://dx.doi.org/10.1136/bmj.j2286

6. Akvardar Y, Demiral Y, Ergor G, Ergor A. Substance use among medical students and physicians in a medical school in Turkey. *Social Psychiatry and Psychiatric Epidemiology: The International Journal for Research in Social and Genetic Epidemiology and Mental Health Services*. 2004;39(6):502-506. doi:https://dx.doi.org/10.1007/s00127-004-0765-1

7. Al-Ghunaim TA, Johnson J, Biyani CS, Alshahrani KM, Dunning A, O'Connor DB. Surgeon burnout, impact on patient safety and professionalism: A systematic review and meta-analysis. *American journal of surgery*. 2022;224(1 Pt A):228-238. doi:https://dx.doi.org/10.1016/j.amjsurg.2021.12.027

8. Alam A, Kurdyak P, Klemensberg J, Griesman J, Bell CM. The characteristics of psychiatrists disciplined by professional colleges in Canada. *PLoS ONE*. 2012;7(11)doi:https://dx.doi.org/10.1371/journal.pone.0050558

9. Albuquerque J, Deshauer D. Physician health: Beyond work-life balance. *Canadian Medical Association Journal*. 2014;186(13):E502-E503. doi:https://dx.doi.org/10.1503/cmaj.140708

10. Alhomayed N, Alhadi A, Alsuhaibani A, et al. Doctors' attitudes toward becoming mentally Ill in Saudi Arabia: Disclosure and treatment preferences. *European Psychiatry*. 2018;48(Supplement 1):S427. doi:https://dx.doi.org/10.1016/j.eurpsy.2017.12.023

11. American College of Emergency P. Physician impairment. Policy statement. *Annals of emergency medicine*. 2014;63(4):502-503.

12. Anfang SA, Faulkner LR, Fromson JA, Gendel MH. The American Psychiatric Association's resource document on guidelines for psychiatric fitness-for-duty evaluations of physicians. *The journal of the American Academy of Psychiatry and the Law*. 2005;33(1):85-8.

13. Angelos P. How Should Colleagues Respond to Diminishing Capacities of an Aging Surgeon? *AMA journal of ethics*. 2016;18(10):986-992. doi:https://dx.doi.org/10.1001/journalofethics.2016.18.10.ecas2-1610

14. Angres D, Delisi S, Alem D, Williams BW. A programmatic approach to treating physicians with a dual diagnosis. *Psychiatric Annals*. 2004;34(10):776-780. doi:https://dx.doi.org/10.3928/0048-5713-20041001-17

15. Angres DH, John A, Bettinardi-Angres K, Agarwal G. The forensic evaluation and rehabilitation of the impaired physician. *Psychiatric Annals*. 2019;49(11):487-491. doi:https://dx.doi.org/10.3928/00485713-20191009-01

16. Angres DH, McGovern MP, Rawal P, Shaw M. Psychiatric comorbidity and physicians with substance use disorders: Clinical characteristics, treatment experiences, and post-treatment functioning. *Addictive Disorders and their Treatment*. 2002;1(3):89-98. doi:https://dx.doi.org/10.1097/00132576-200209000-00003

17. Angres DH, McGovern MP, Shaw MF, Rawal P. Psychiatric Comorbidity and Physicians with Substance Use Disorders: A Comparison between the 1980s and 1990s. *Journal of Addictive Diseases*. 2003;22(3):79-87. doi:https://dx.doi.org/10.1300/J069v22n03_07

18. Anonymous. Statewide physician health program. *Wisconsin medical journal*. 1994;93(7):345-6.

19. Anonymous. Statewide Physician Health Program. *Wisconsin medical journal*. 1995;94(7):384-5.

20. Anonymous. American College of Emergency Physicians: physician impairment. *Annals of emergency medicine*. 1995;25(2):307.

21. Anonymous. Statewide physician health program. *Wisconsin medical journal*. 1996;95(7):471-2.

22. Anonymous. The impaired physician. The Ethics Committee of the American Academy of Otolaryngology-Head and Neck Surgery. *Otolaryngology--head and neck surgery : official journal of American Academy of Otolaryngology-Head and Neck Surgery*. 1996;115(3):213-9.

23. Anonymous. Courts deal with impaired physician issues. Altman v. New York City Health and Hospitals Corp.; Ambrosino v. Metropolitan Life Insurance Co. *Hospital law newsletter*. 1996;13(9):3-8.

24. Anonymous. When staff members become substance abusers: identifying the drug-impaired clinician. *ED management : the monthly update on emergency department management*. 1997;9(12):145-7.

25. Anonymous. Statewide Physician Health Program. *Wisconsin Medical Journal*. 2000;99(4):32-33.

26. Anonymous. Drug-impaired doctors. *South African Medical Journal*. 2004;94(9):711.

27. Anonymous. Statement on the aging surgeon. *Bulletin of the American College of Surgeons*. 2016;101(1):42-3.

28. Anonymous. ACOG Committee Opinion: The late-career obstetrician-gynecologist. *Obstetrics and Gynecology*. 2018;131(6):E200-E204. doi:https://dx.doi.org/10.1097/AOG.0000000000002642

29. Antunes MJ. The Age of The Surgeon: How Old Is Too Old? *Acta medica portuguesa*. 2019;32(6):413-414. doi:https://dx.doi.org/10.20344/amp.12160

30. Armstrong KA, Reynolds EE. Opportunities and Challenges in Valuing and Evaluating Aging Physicians. *JAMA*. 2020;323(2):125-126. doi:https://dx.doi.org/10.1001/jama.2019.19706

31. Asserson DB, Janis JE. The Aging Surgeon: Evidence and Experience. *Aesthetic Surgery Journal*. 2022;42(1):121-127. doi:https://dx.doi.org/10.1093/asj/sjab145

32. Austin EE, Do V, Nullwala R, et al. Systematic review of the factors and the key indicators that identify doctors at risk of complaints, malpractice claims or impaired performance. *BMJ open*. 2021;11(8):e050377. doi:https://dx.doi.org/10.1136/bmjopen-2021-050377

33. Avery, Daniel, McCormick. The impaired physician. *Primary care update for Ob/Gyns*. 2000;7(4):154-160.

34. Babu MA, Liau LM, Spinner RJ, Meyer FB. Maintenance of certification and the aging neurosurgeon. *Clinical Neurosurgery*. 2016;63(Supplement 1):194-195. doi:https://dx.doi.org/10.1227/01.neu.0000489814.98347.ee

35. Babu MA, Liau LM, Spinner RJ, Meyer FB. The Aging Neurosurgeon: When Is Enough, Enough? Attitudes Toward Ceasing Practice and Testing in Late Career. *Mayo Clinic proceedings*. 2017;92(12):1746-1752. doi:https://dx.doi.org/10.1016/j.mayocp.2017.09.004

36. Baldwin DC, Jr., Daugherty SR, Rowley BD. Unethical and unprofessional conduct observed by residents during their first year of training. *Academic medicine : journal of the Association of American Medical Colleges*. 1998;73(11):1195-200.

37. Barden N, Caleb R. Fitness to practise. *The handbook of professional, ethical and research practice for psychologists, counsellors, psychotherapists and psychiatrists, 3rd ed*. 2020;(Barden, N. (2001) The responsibility of the supervisor in the British Association for Counselling and Psychotherapy's codes of ethics and practice. In S.Wheeler & D.King (Eds.). Supervising Counsellors: Issues of Responsibility, London: Sage, pp. 41-52.Bo):105-116. doi:https://dx.doi.org/10.4324/9780429428838-9

38. Barrigar DL, Flagel DC, Upshur REG. Hepatitis B virus infected physicians and disclosure of transmission risks to patients: A critical analysis. *BMC Medical Ethics*. 2001;2(ABPC. (1991). The APIC statement on purposes for and elements of patient notification programs related to the health care worker infected with the human immunodeficiency virus or the hepatitis B "e" antigen. American Journal of Infection Control 1991, 19:)doi:https://dx.doi.org/10.1186/1472-6939-2-4

39. Bateman EA, Viana R. Burnout among specialists and trainees in physical medicine and rehabilitation: A systematic review. *Journal of rehabilitation medicine*. 2019;51(11):869-874. doi:https://dx.doi.org/10.2340/16501977-2614

40. Baxter AD, Boet S, Reid D, Skidmore G. The aging anesthesiologist: a narrative review and suggested strategies. *Canadian journal of anaesthesia = Journal canadien d'anesthesie*. 2014;61(9):865-75. doi:https://dx.doi.org/10.1007/s12630-014-0194-x

41. Becker GJ. Understanding and applying the principles of contemporary medical professionalism: Illustration of a suggested approach, part 1. *Journal of the American College of Radiology*. 2014;11(12):1110-1112. doi:https://dx.doi.org/10.1016/j.jacr.2014.08.025

42. Beekman ATF. Aging Affects Us All: Aging Physicians and Screening for Impaired Professional Proficiency. *The American journal of geriatric psychiatry : official journal of the American Association for Geriatric Psychiatry*. 2018;26(6):641-642. doi:https://dx.doi.org/10.1016/j.jagp.2018.03.006

43. Beierle SP, Kirkpatrick BA, Heidel RE, et al. Evaluating and Exploring Variations in Surgical Resident Emotional Intelligence and Burnout. *Journal of surgical education*. 2019;76(3):628-636. doi:https://dx.doi.org/10.1016/j.jsurg.2018.11.004

44. Beletsky L, Wakeman SE, Fiscella K. Practicing What We Preach - Ending Physician Health Program Bans on Opioid-Agonist Therapy. *The New England journal of medicine*. 2019;381(9):796-798. doi:https://dx.doi.org/10.1056/NEJMp1907875

45. Bell PF, Semelka MW, Bigdeli L. Drug Testing Incoming Residents and Medical Students in Family Medicine Training: A Survey of Program Policies and Practices. *Journal of graduate medical education*. 2015;7(1):59-64. doi:https://dx.doi.org/10.4300/JGME-D-14-00308.1

46. Benatar SR. The impaired doctor. *South African medical journal = Suid-Afrikaanse tydskrif vir geneeskunde*. 1994;84(10):651-2.

47. Bender CE, Heilbrun ME, Truong HB, Bluth EI. The impaired radiologist. *Journal of the American College of Radiology : JACR*. 2015;12(3):302-6. doi:https://dx.doi.org/10.1016/j.jacr.2014.09.021

48. Bennett AJ, Roman B, Arnold LM, Kay J, Goldenhar LM. Professionalism deficits among medical students: models of identification and intervention. *Academic psychiatry : the journal of the American Association of Directors of Psychiatric Residency Training and the Association for Academic Psychiatry*. 2005;29(5):426-32.

49. Benzer DG. Stress impairment in physicians. *WMJ : official publication of the State Medical Society of Wisconsin*. 2001;100(1):20-3.

50. Beran RG. Mandatory notification of impaired doctors. *Internal medicine journal*. 2014;44(12a):1161-5. doi:https://dx.doi.org/10.1111/imj.12604

51. Berliner H. Underperforming doctors. Recovery services. *The Health service journal*. 1999;109(5651):28-9.

52. Beschoner P, Limbrecht-Ecklundt K, Jerg-Bretzke L. [Mental health among physicians : Burnout, depression, anxiety and substance abuse in the occupational context]. *Der Nervenarzt*. 2019;90(9):961-974. doi:https://dx.doi.org/10.1007/s00115-019-0739-x

53. Blair Carlson H, Dilts SL, Radcliff S. Physicians with substance abuse problems and their recovery environment: A survey. *Journal of Substance Abuse Treatment*. 1994;11(2):113-119. doi:https://dx.doi.org/10.1016/0740-5472%2894%2990027-2

54. Blasier RB. The problem of the aging surgeon: when surgeon age becomes a surgical risk factor. *Clinical orthopaedics and related research*. 2009;467(2):402-11. doi:https://dx.doi.org/10.1007/s11999-008-0587-7

55. Blondell RD. Taking a proactive approach to physician impairment. *Postgraduate medicine*. 2005;118(1):16-8.

56. Bogowicz P, Ferguson J, Gilvarry E, Kamali F, Kaner E, Newbury-Birch D. Alcohol and other substance use among medical and law students at a UK university: a cross-sectional questionnaire survey. *Postgraduate medical journal*. 2018;94(1109):131-136. doi:https://dx.doi.org/10.1136/postgradmedj-2017-135136

57. Bohigian GM, Bondurant R, Croughan J. The impaired and disruptive physician: the Missouri Physicians' Health Program--an update (1995-2002). *Journal of addictive diseases*. 2005;24(1):13-23.

58. Bohigian GM, Croughan JL, Bondurant R. Substance abuse and dependence in physicians: the Missouri Physicians Health Program--an update (1995-2001). *Missouri medicine*. 2002;99(4):161-5.

59. Bohigian GM, Croughan JL, Sanders K. The impaired physician, Part II: Missouri State Physicians Health Program. *Missouri medicine*. 1994;91(6):275-7.

60. Bohigian GM, Croughan JL, Sanders K, Evans ML, Bondurant R, Platt C. Substance abuse and dependence in physicians: the Missouri Physicians' Health Program. *Southern medical journal*. 1996;89(11):1078-80.

61. Boisaubin EV. Causes and treatment of impairment and burnout in physicians: The epidemic within. *Faculty health in academic medicine: Physicians, scientists, and the pressures of success*. 2009;(American Medical Association Council on Mental Health. (1973) The sick physician: impairment by psychiatric disorders including alcoholism and drug dependency Journal of the American Medical Association 223, 684-687.Boisaubin, E. V., & Levine, R. E. (2001):29-38. doi:https://dx.doi.org/10.1007/978-1-60327-451-7_3

62. Boisaubin EV, Levine RE. Identifying and assisting the impaired physician. *The American journal of the medical sciences*. 2001;322(1):31-6.

63. Bosch X. First impaired physicians therapy program appears to be successful in Spain. *JAMA*. 2000;283(24):3186-7.

64. Bouaziz H, Yavordios PG. The physician, second victim? *Praticien en Anesthesie Reanimation*. 2020;24(6):329-332. doi:https://dx.doi.org/10.1016/j.pratan.2020.08.004

65. Boyd JW. A Call for National Standards and Oversight of State Physician Health Programs. *Journal of addiction medicine*. 2015;9(6):431-2. doi:https://dx.doi.org/10.1097/ADM.0000000000000174

66. Boyd JW. Deciding whether to refer a colleague to a physician health program. *AMA journal of ethics*. 2015;17(10):888-93. doi:https://dx.doi.org/10.1001/journalofethics.2015.17.10.spec1-1510

67. Boyd JW. Substance use and addictive behaviors among physicians. *Physician mental health and well-being: Research and practice*. 2017;(Agius, R., Kintz, P., & European Workplace Drug Testing Society. (2010). Guidelines for European workplace drug and alcohol testing in hair. Drug Test Anal. 2010, 2(8).367-76.Baldisseri, M. R. (2007). Impaired healthcare professional. Crit Care Med. 2007;):177-193. doi:https://dx.doi.org/10.1007/978-3-319-55583-6_8

68. Boyd JW, Knight JR. Ethical and managerial considerations regarding state physician health programs. *Journal of addiction medicine*. 2012;6(4):243-6. doi:https://dx.doi.org/10.1097/ADM.0b013e318262ab09

69. Boyd W. State physician health programs require national standards and external oversight. *African Journal of Psychiatry (South Africa)*. 2016;19(1):1000346. doi:https://dx.doi.org/10.4172/2378-5756.1000346

70. Bradfield O, Jenkins K, Spittal M, Bismark M. Australian and New Zealand doctors' experiences of disciplinary notifications, investigations, proceedings and interventions relating to alleged mental health impairment: a qualitative analysis of interviews. *International journal of law and psychiatry*. 2023;86(grp, 7806862):101857. doi:https://dx.doi.org/10.1016/j.ijlp.2022.101857

71. Bradfield OM, Spittal MJ, Bismark MM. Regulation in Need of Therapy? Analysis of Regulatory Decisions Relating to Impaired Doctors from 2010 to 2020. *Journal of law and medicine*. 2022;29(4):1090-1108.

72. Bradley J. Fitness to practise and the spectre of erasure: Some reflections from a psychiatrist assessor. *The Medico-legal journal*. 2022;90(4):212-215. doi:https://dx.doi.org/10.1177/00258172221113973

73. Brannigan L, Beeton A. The impaired clinician and the health and wellness of practitioners in anaesthesia and critical care. *Southern African Journal of Anaesthesia and Analgesia*. 2013;19(3):137. doi:http://dx.doi.org/10.1080/22201173.2013.10872912

74. Bransi A, Winter L, Glahn A, Kahl KG. Addictive disorders in physicians. *Der Nervenarzt*. 2020;91(1):77-90. doi:https://dx.doi.org/10.1007/s00115-019-00854-3

75. Braquehais MD, Arrizabalaga P, Lusilla P, et al. Gender differences in demographic and clinical features of physicians admitted to a program for medical professionals with mental disorders. *Frontiers in Psychiatry*. 2016;7(Alonso, J, Angermeyer, MC, Bernet, S, Bruffaerts, R, Brugha, TS, & Brison, H, et al Prevalence of mental disorders in Europe: results from the European Study of Epidemiology of Mental Disorders (ESEMeD) project. Acta Psychiatr Scand Suppl (2004) 109(420):)doi:https://dx.doi.org/10.3389/fpsyt.2016.00181

76. Braquehais MD, Tresidder A, DuPont RL. Service provision to physicians with mental health and addiction problems. *Current opinion in psychiatry*. 2015;28(4):324-9. doi:https://dx.doi.org/10.1097/YCO.0000000000000166

77. Braquehais MD, Vargas-Caceres S, Nieva G, et al. Characteristics of resident physicians accessing a specialised mental health service: a retrospective study. *BMJ open*. 2021;11(12):e055184. doi:https://dx.doi.org/10.1136/bmjopen-2021-055184

78. Breen KJ, Court JM, Katsoris J. Impaired doctors. The modern approach of medical boards. *Australian family physician*. 1998;27(11):1005-8.

79. Brewster JM. "Physicians' experiences with impaired colleagues": Comment. *JAMA: Journal of the American Medical Association*. 2010;304(17):1896. doi:https://dx.doi.org/10.1001/jama.2010.1559

80. Brewster JM, Kaufmann IM, Hutchison S, MacWilliam C. Characteristics and outcomes of doctors in a substance dependence monitoring programme in Canada: prospective descriptive study. *BMJ (Clinical research ed)*. 2008;337(8900488, bmj, 101090866):a2098. doi:https://dx.doi.org/10.1136/bmj.a2098

81. Brooke D. The addicted doctor: Caring professionals? *The British Journal of Psychiatry*. 1995;166(2):149-153. doi:https://dx.doi.org/10.1192/bjp.166.2.149

82. Brooks E, Early SR, Gundersen DC, Shore JH, Gendel MH. Comparing substance use monitoring and treatment variations among physician health programs. *The American journal on addictions*. 2012;21(4):327-34. doi:https://dx.doi.org/10.1111/j.1521-0391.2012.00239.x

83. Brooks E, Gendel MH, Gundersen DC, et al. Physician health programmes and malpractice claims: reducing risk through monitoring. *Occupational medicine (Oxford, England)*. 2013;63(4):274-80. doi:https://dx.doi.org/10.1093/occmed/kqt036

84. Brooks E, Gendel MH, Parry AL, Humphreys S, Early SR. Challenging cognitive cases among physician populations: case vignettes and recommendations. *Occupational medicine (Oxford, England)*. 2017;67(1):68-70. doi:https://dx.doi.org/10.1093/occmed/kqw132

85. Brooks F, Osborn CJ. Counseling Impaired Professionals. *Critical incidents in addictions counseling*. 2005;(DiClemente, C. C. (2003). Addiction and change: How addictions develop and addicted people recover. New York: Guilford Press. 2003-00572-000.Knapp, C. (1996). Drinking: A love story. New York: Dial Press.McGovern, M. P., Angres, D. H., Shaw, M., & Rawal):1-8.

86. Brower KJ, Riba MB. Physician mental health and well-being: Research and practice. *Physician mental health and well-being: Research and practice*. 2017;doi:https://dx.doi.org/10.1007/978-3-319-55583-6

87. Brown SD, Goske MJ, Johnson CM. Beyond substance abuse: stress, burnout, and depression as causes of physician impairment and disruptive behavior. *Journal of the American College of Radiology : JACR*. 2009;6(7):479-85. doi:https://dx.doi.org/10.1016/j.jacr.2008.11.029

88. Browning GG. Who will have the 'guts' to quantify the decline in surgical skills of older surgeons? A commentary on the paper that shows that this occurs in thyroid surgeons. *Clinical otolaryngology : official journal of ENT-UK ; official journal of Netherlands Society for Oto-Rhino-Laryngology & Cervico-Facial Surgery*. 2012;37(2):147. doi:https://dx.doi.org/10.1111/j.1749-4486.2012.02468.x

89. Bruguera E, Heredia M, Llavayol E, et al. Integral Treatment Programme for Addicted Physicians: Results from The Galatea Care Programme for Sick Physicians. *European addiction research*. 2020;26(3):122-130. doi:https://dx.doi.org/10.1159/000505914

90. Bryson EO. The impaired anesthesiologist: where do we draw the line? *Journal of clinical anesthesia*. 2010;22(5):311-2. doi:https://dx.doi.org/10.1016/j.jclinane.2010.02.004

91. Buhl A, Oreskovich MR, Meredith CW, Campbell MD, Dupont RL. Prognosis for the recovery of surgeons from chemical dependency: a 5-year outcome study. *Archives of surgery (Chicago, Ill : 1960)*. 2011;146(11):1286-91. doi:https://dx.doi.org/10.1001/archsurg.2011.271

92. Burroughs J. Dealing with the aging physician advocacy or betrayal? *Physician executive*. 2012;38(6):38-41.

93. Campbell G, Rollin AM, Smith AF. Cases relating to anaesthetists handled by the UK General Medical Council in 2009: methodological approach and patterns of referral. *Anaesthesia*. 2013;68(5):453-60. doi:https://dx.doi.org/10.1111/anae.12117

94. Candilis PJ. Physician Health Programs and the Social Contract. *AMA journal of ethics*. 2016;18(1):77-81. doi:https://dx.doi.org/10.1001/journalofethics.2016.18.1.corr1-1601

95. Candilis PJ, Kim DT, Sulmasy LS. Physician Impairment and Rehabilitation: Reintegration Into Medical Practice While Ensuring Patient Safety: A Position Paper From the American College of Physicians. *Annals of internal medicine*. 2019;170(12):871-879. doi:https://dx.doi.org/10.7326/M18-3605

96. Cappell MS. Two case reports of novel syndrome of bizarre performance of gastrointestinal endoscopy due to toxic encephalopathy of endoscopists among 181767 endoscopies in a 13-year-university hospital review: Endoscopists, first do no harm! *World journal of gastroenterology*. 2020;26(9):984-991. doi:https://dx.doi.org/10.3748/wjg.v26.i9.984

97. Carinci AJ, Christo PJ. Physician impairment: is recovery feasible? *Pain physician*. 2009;12(3):487-91.

98. Carpenter WR, Wieberg J, Johns H. The Missouri Physician and Health Professional Wellness Program: A Comprehensive Resource for Physician Wellness. *Missouri medicine*. 2021;118(1):41-44.

99. Carr GD. Our hospital is revising language to address "physician impairment". What role does the Mississippi Recovering Physicians Program (MRPP) play? *Journal of the Mississippi State Medical Association*. 2002;43(10):325.

100. Carr GD, Hall PB, Finlayson AJR, DuPont RL. Physician health programs: The US model. *Physician mental health and well-being: Research and practice*. 2017;(AbuDagga, A., Wolfe, S. M., Carome, M., & Oshel, R. E. (2016). Cross-sectional analysis of the 1039 US physicians reponed to the National Practitioner Data Bank for sexual misconduct, 2003-2013. PLoS One. 2016; 11(2) e0147800AMA Council on Ethical and Jud):265-294. doi:https://dx.doi.org/10.1007/978-3-319-55583-6_12

101. Casey G, Lemay K, Ji J, et al. Medico-legal cases associated with older physicians' cognitive ability to practice medicine. *Journal of healthcare risk management : the journal of the American Society for Healthcare Risk Management*. 2023;(bm7, 9305245)doi:https://dx.doi.org/10.1002/jhrm.21562

102. Centrella M. Physician addiction and impairment--current thinking: a review. *Journal of addictive diseases*. 1994;13(1):91-105.

103. Chai C-Y, Chen C-H, Lin H-W, Lin H-C. Association of increasing surgeon age with decreasing in-hospital mortality after coronary artery bypass graft surgery. *World journal of surgery*. 2010;34(1):3-9. doi:https://dx.doi.org/10.1007/s00268-009-0291-0

104. Chase-Lubitz JF. Legal Issues and the Aging Physician. *Rhode Island medical journal (2013)*. 2017;100(9):23-25.

105. Chen JH, Rosengart TK. Commentary: Ensuring life-long competency of early-career and late-career surgeons. *The Journal of thoracic and cardiovascular surgery*. 2023;(k9j, 0376343)doi:https://dx.doi.org/10.1016/j.jtcvs.2023.10.016

106. Cherry DP, 3rd, Aufderheide DH. Care of prison inmates by impaired disciplined physicians. *JAMA*. 1999;281(20):1889-1.

107. Clay SW, Conatser RR. Characteristics of physicians disciplined by the State Medical Board of Ohio. *Journal of the American Osteopathic Association*. 2003;103(2):81-88.

108. Collier R. Diagnosing the aging physician. *CMAJ : Canadian Medical Association journal = journal de l'Association medicale canadienne*. 2008;178(9):1121-3. doi:https://dx.doi.org/10.1503/cmaj.080470

109. Collier S. Invited perspective on "unsolicited patient complaints identify physicians with evidence of neurocognitive disorders". *The American Journal of Geriatric Psychiatry*. 2018;26(9):937-938. doi:https://dx.doi.org/10.1016/j.jagp.2018.05.006

110. Cook RJ, Dickens BM. Patient care and the health-impaired practitioner. *International Journal of Gynecology and Obstetrics*. 2002;78(2):171-177. doi:https://dx.doi.org/10.1016/S0020-7292%2802%2900095-4

111. Coombs RH. Drug-impaired professionals. *Drug-impaired professionals*. 1997;

112. Cooney L, Balcezak T. Cognitive Testing of Older Clinicians Prior to Recredentialing. *JAMA*. 2020;323(2):179-180. doi:https://dx.doi.org/10.1001/jama.2019.18665

113. Cooper WO, Martinez W, Domenico HJ, et al. Unsolicited Patient Complaints Identify Physicians with Evidence of Neurocognitive Disorders. *The American journal of geriatric psychiatry : official journal of the American Association for Geriatric Psychiatry*. 2018;26(9):927-936. doi:https://dx.doi.org/10.1016/j.jagp.2018.04.005

114. Cottler LB, Ajinkya S, Merlo LJ, Nixon SJ, Ben Abdallah A, Gold MS. Lifetime psychiatric and substance use disorders among impaired physicians in a physicians health program: comparison to a general treatment population: psychopathology of impaired physicians. *Journal of addiction medicine*. 2013;7(2):108-12. doi:https://dx.doi.org/10.1097/ADM.0b013e31827fadc9

115. Cullen MJ, Konia MR, Borman-Shoap EC, et al. Not all unprofessional behaviors are equal: The creation of a checklist of bad behaviors. *Medical teacher*. 2017;39(1):85-91. doi:https://dx.doi.org/10.1080/0142159X.2016.1231917

116. Cummings SM, Merlo L, Cottler L. Mechanisms of prescription drug diversion among impaired physicians. *Journal of addictive diseases*. 2011;30(3):195-202. doi:https://dx.doi.org/10.1080/10550887.2011.581962

117. Cunningham W, Cookson T. Addressing stress-related impairment in doctors. A survey of providers' and doctors' experience of a funded counselling service in New Zealand. *The New Zealand medical journal*. 2009;122(1300):19-28.

118. Czernichow S, Bonnet F. Drug abuse among anesthesiologist. *Annales Francaises d'Anesthesie et de Reanimation*. 2000;19(9):668-674. doi:https://dx.doi.org/10.1016/S0750-7658%2800%2900295-1

119. David TJ, Ellson S. Refusal to grant provisional General Medical Council registration to U.K. medical graduates. *The Medico-legal journal*. 2015;83(3):142-6. doi:https://dx.doi.org/10.1177/0025817215579169

120. Del Bene VA, Brandt J. Identifying neuropsychologically impaired physicians. *The Clinical neuropsychologist*. 2020;34(2):318-331. doi:https://dx.doi.org/10.1080/13854046.2019.1666922

121. Del Bene VA, Geldmacher DS, Howard G, et al. A rationale and framework for addressing physician cognitive impairment. *Frontiers in public health*. 2023;11(101616579):1245770. doi:https://dx.doi.org/10.3389/fpubh.2023.1245770

122. Dellinger EP, Pellegrini CA, Gallagher TH. The Aging Physician and the Medical Profession: A Review. *JAMA surgery*. 2017;152(10):967-971. doi:https://dx.doi.org/10.1001/jamasurg.2017.2342

123. Devi G. Alzheimer's disease in physicians -Assessing professional competence and tempering stigma. *New England Journal of Medicine*. 2018;378(12):1073-1075. doi:https://dx.doi.org/10.1056/NEJMp1716381

124. Devi G, Gitelman DR, Press D, Daffner KR. Cognitive Impairment in Aging Physicians: Current Challenges and Possible Solutions. *Neurology Clinical practice*. 2021;11(2):167-174. doi:https://dx.doi.org/10.1212/CPJ.0000000000000829

125. Dilts SL, Gendel M, Lepoff R, Clark C, Radcliff S. The Colorado Physician Health Program. Observations at 7 years. *American Journal on Addictions*. 1994;3(4):337-345.

126. Donaghy R, Tomatsu S, Kerns P, White C, Ratliff J. An Educational Workshop to Improve Neurology Resident Understanding of Burnout, Substance Abuse, and Mood Disorders. *MedEdPORTAL : the journal of teaching and learning resources*. 2021;17(101714390):11164. doi:https://dx.doi.org/10.15766/mep_2374-8265.11164

127. DuBois JM, Walsh HA, Chibnall JT, et al. Sexual Violation of Patients by Physicians: A Mixed-Methods, Exploratory Analysis of 101 Cases. *Sexual abuse : a journal of research and treatment*. 2019;31(5):503-523. doi:https://dx.doi.org/10.1177/1079063217712217

128. DuPont RL. Opioid use disorder in physicians. *New England Journal of Medicine*. 2019;381(23):2279-2280. doi:https://dx.doi.org/10.1056/NEJMc1913323

129. DuPont RL, McLellan AT, Carr G, Gendel M, Skipper GE. How are addicted physicians treated? A national survey of Physician Health Programs. *Journal of substance abuse treatment*. 2009;37(1):1-7. doi:https://dx.doi.org/10.1016/j.jsat.2009.03.010

130. DuPont RL, Seppala MD, White WL. The three missing elements in the treatment of substance use disorders: Lessons from the physician health programs. *Journal of addictive diseases*. 2016;35(1):3-7. doi:https://dx.doi.org/10.1080/10550887.2015.1102797

131. Durning SJ, Holmboe E, Lipner R. Aging physicians: some lessons from the literature outside of medicine and potential implications for practice. *Maryland medicine : MM : a publication of MEDCHI, the Maryland State Medical Society*. 2013;14(3):11-16.

132. Dyrbye LN, West CP, Hunderfund AL, et al. Relationship Between Burnout, Professional Behaviors, and Cost-Conscious Attitudes Among US Physicians. *Journal of general internal medicine*. 2020;35(5):1465-1476. doi:https://dx.doi.org/10.1007/s11606-019-05376-x

133. Dyrbye LN, West CP, Satele D, Boone S, Sloan J, Shanafelt TD. A national study of medical students' attitudes toward self-prescribing and responsibility to report impaired colleagues. *Academic medicine : journal of the Association of American Medical Colleges*. 2015;90(4):485-93. doi:https://dx.doi.org/10.1097/ACM.0000000000000604

134. Eisenmann N. Differences between Licensed Healthcare Professionals with Substance Use-Related Licensure Discipline. *Substance use & misuse*. 2020;55(12):2035-2042. doi:https://dx.doi.org/10.1080/10826084.2020.1788090

135. Elliott HW, Arnold EM, Brenes GA, Silvia L, Rosenquist PB. Attention deficit hyperactivity disorder accommodations for psychiatry residents. *Academic Psychiatry*. 2007;31(4):290-296. doi:https://dx.doi.org/10.1176/appi.ap.31.4.290

136. Eva KW. The aging physician: Changes in cognitive processing and their impact on medical practice. *Academic Medicine*. 2002;77(10 SUPPL.):S1-S6. doi:https://dx.doi.org/10.1097/00001888-200210001-00002

137. Farber NJ, Gilibert SG, Aboff BM, Collier VU, Weiner J, Boyer EG. Physicians' willingness to report impaired colleagues. *Social science & medicine (1982)*. 2005;61(8):1772-5.

138. Finlayson AJR, Dietrich MS, Neufeld R, Roback H, Martin PR. Restoring professionalism: the physician fitness-for-duty evaluation. *General hospital psychiatry*. 2013;35(6):659-63. doi:https://dx.doi.org/10.1016/j.genhosppsych.2013.06.009

139. Finlayson AJR, Iannelli RJ, Brown KP, Neufeld RE, DuPont RL, Campbell MD. Re: physician suicide and physician health programs. *General hospital psychiatry*. 2016;40(fnk, 7905527):84-5. doi:https://dx.doi.org/10.1016/j.genhosppsych.2016.01.001

140. Finlayson AJR, Kim A, Mallory AB, Vandekar S, Martin PR. Changing characteristics of physicians referred for fitness-for-duty evaluation. *General hospital psychiatry*. 2022;77(fnk, 7905527):128-129. doi:https://dx.doi.org/10.1016/j.genhosppsych.2022.05.008

141. Firth-Cozens J. Interventions to improve physicians' well-being and patient care. *Social Science & Medicine*. 2001;52(2):215-222. doi:https://dx.doi.org/10.1016/S0277-9536%2800%2900221-5

142. Fletcher I, Castle M, Scarpa A, Myers O, Lawrence E. An exploration of medical student attitudes towards disclosure of mental illness. *Medical education online*. 2020;25(1):1727713. doi:https://dx.doi.org/10.1080/10872981.2020.1727713

143. Flowers WM, Jr. Sometimes doctors need help too--the Mississippi Recovering Physicians Program. *Journal of the Mississippi State Medical Association*. 1999;40(7):252-5.

144. Fost N, Chander K, Miles SH. Licensing boards and the stigma of mental illness. *JAMA: Journal of the American Medical Association*. 1999;281(7):606-607. doi:https://dx.doi.org/10.1001/jama.281.7.606

145. Fowlie DG. The misuse of alcohol and other drugs by doctors: A UK report and one region's response. *Alcohol and Alcoholism*. 1999;34(5):666-671. doi:https://dx.doi.org/10.1093/alcalc/34.5.666

146. Fowlie DG. Invited commentary: Doctors' drinking and fitness to practise. *Alcohol and Alcoholism*. 2005;40(6):483-484. doi:https://dx.doi.org/10.1093/alcalc/agh218

147. Fox DM. Commentary: Impaired physicians and the new politics of accountability. *Academic Medicine*. 2009;84(6):692-694. doi:https://dx.doi.org/10.1097/ACM.0b013e3181a402e6

148. Frazer A, Tanzer M. Hanging up the surgical cap: Assessing the competence of aging surgeons. *World journal of orthopedics*. 2021;12(4):234-245. doi:https://dx.doi.org/10.5312/wjo.v12.i4.234

149. Freedman JL, Crow FF, Gutheil TG, Sanchez LT, Suzuki J. Treating a physician patient with psychosis. *Asian Journal of Psychiatry*. 2012;5(2):193-198. doi:https://dx.doi.org/10.1016/j.ajp.2012.03.001

150. Frei M, Wile C, Jenkins K, Whelan G. Who cares for addicted doctors? Reviewing an Australian program for impaired medical practitioners. *Internal Medicine Journal*. 2010;40(SUPPL. 1):17. doi:https://dx.doi.org/10.1111/j.1445-5994.2010.02186.x

151. Galanter M, Dermatis H, Mansky P, McIntyre J, Perez-Fuentes G. Substance-abusing physicians: monitoring and twelve-step-based treatment. *The American journal on addictions*. 2007;16(2):117-23.

152. Ganapathy K. Should neurosurgeons retire? *Neurology India*. 2019;67(2):370-374. doi:https://dx.doi.org/10.4103/0028-3886.258036

153. Ganley OH, Pendergast WJ, Wilkerson MW, Mattingly DE. Outcome study of substance impaired physicians and physician assistants under contract with North Carolina Physicians Health Program for the period 1995-2000. *Journal of addictive diseases*. 2005;24(1):1-12.

154. Garrett K, Kaups KL. The aging surgeon: when is it time to leave active practice? *Bulletin of the American College of Surgeons*. 2014;99(4):32-5.

155. Garrett KD, Perry W, Williams B, Korinek L, Bazzo DEJ. Cognitive Screening Tools for Late Career Physicians: A Critical Review. *Journal of geriatric psychiatry and neurology*. 2021;34(3):171-180. doi:https://dx.doi.org/10.1177/0891988720924712

156. Gaudet CE, Del Bene VA. Neuropsychological Assessment of the Aging Physician: A Review & Commentary. *Journal of geriatric psychiatry and neurology*. 2022;35(3):271-279. doi:https://dx.doi.org/10.1177/08919887211016063

157. Gautham KS. Addressing Disruptive and Unprofessional Physician Behavior. *Joint Commission Journal on Quality and Patient Safety*. 2020;46(2):61-63. doi:https://dx.doi.org/10.1016/j.jcjq.2019.12.002

158. Gendel MH. Treatment adherence in physicians. *Primary Psychiatry*. 2005;12(6):48-54.

159. Gentile JP. Mandated psychotherapy with the impaired physician. *Psychiatry (Edgmont (Pa : Township))*. 2008;5(2):42-9.

160. Gerrity MS. Interventions to improve physicians' well-being and patient care: A commentary. *Social Science & Medicine*. 2001;52(2):223-225. doi:https://dx.doi.org/10.1016/S0277-9536%2800%2900222-7

161. Geuijen PM, Pars E, Kuppens JM, et al. Barriers and Facilitators to Seek Help for Substance Use Disorder among Dutch Physicians: A Qualitative Study. *European addiction research*. 2022;28(1):23-32. doi:https://dx.doi.org/10.1159/000517043

162. Glassman R, Libman H, Matsumoto E. Breaking bad: The importance of recognizing physicians impaired by substance abuse disorders. *Journal of General Internal Medicine*. 2014;29(SUPPL. 1):S326.

163. Gold KJ, Andrew LB, Goldman EB, Schwenk TL. "I would never want to have a mental health diagnosis on my record": A survey of female physicians on mental health diagnosis, treatment, and reporting. *General hospital psychiatry*. 2016;43(fnk, 7905527):51-57. doi:https://dx.doi.org/10.1016/j.genhosppsych.2016.09.004

164. Gold KJ, Shih ER, Goldman EB, Schwenk TL. Do US Medical Licensing Applications Treat Mental and Physical Illness Equivalently? *Family medicine*. 2017;49(6):464-467.

165. Gold MS. Physician health and impairment. *Psychiatric Annals*. 2004;34(10):736-741. doi:https://dx.doi.org/10.3928/0048-5713-20041001-04

166. Gold MS, Gres K, Frost-Pineda K. Drug- and Alcohol-impaired Physicians: Current Research and Progress. *Directions in Psychiatry*. 2006;26(2):117-128.

167. Goldberg R, Thomas H, Penner L. Issues of concern to emergency physicians in pre-retirement years: A survey. *Journal of Emergency Medicine*. 2011;40(6):706-713. doi:https://dx.doi.org/10.1016/j.jemermed.2009.08.020

168. Goldenberg MDO, Miotto KMD, Skipper GEMD, Sanford JBA. Outcomes of Physicians with Substance Use Disorders in State Physician Health Programs: A Narrative Review. *Journal of psychoactive drugs*. 2020;52(3):195-202. doi:https://dx.doi.org/10.1080/02791072.2020.1734696

169. Goldman LS. Physician impairment and health: a brief overview. *South Dakota journal of medicine*. 1997;50(10):359-60.

170. Goldman LS. Physician impairment and health: a brief overview. *Alaska medicine*. 1997;39(3):80-1.

171. Goldman LS. Physician impairment and health: a brief overview. *The Journal of the Kentucky Medical Association*. 1998;96(1):25-6.

172. Gosselin MM, Alolabi B, Dickens JF, et al. Cross-Sectional Survey Results on Mental Health Among Orthopedic Surgery Residents Across North America. *Journal of surgical education*. 2019;76(6):1484-1491. doi:https://dx.doi.org/10.1016/j.jsurg.2019.06.003

173. Gotlib D, Lemmen C. Distinguishing physician misconduct from physician disability. *Journal of the American Academy of Psychiatry and the Law*. 2016;44(4):499-501.

174. Gotlib D, Lemmen C. "Distinguishing physician misconduct from physician disability": Erratum. *Journal of the American Academy of Psychiatry and the Law*. 2018;46(4):556.

175. Graham C. Poland wrestles with problem of drunken doctors. *The Lancet*. 2006;368(9531):190-191. doi:https://dx.doi.org/10.1016/S0140-6736%2806%2969020-X

176. Grandjean B, Grell C. Why No Mandatory Retirement Age Exists for Physicians: Important Lessons for Employers. *Missouri medicine*. 2019;116(5):357-360.

177. Grant A, Rix A, Shrewsbury D. 'If you're crying this much you shouldn't be a consultant': the lived experience of UK doctors in training with mental illness. *International review of psychiatry (Abingdon, England)*. 2019;31(7-8):673-683. doi:https://dx.doi.org/10.1080/09540261.2019.1586326

178. Gray RW. TMF broadens scope, continues work with impaired physicians. *Tennessee medicine : journal of the Tennessee Medical Association*. 2011;104(5):35.

179. Greenwood MJ, Beasley-Greenwood M. Medical licensure and credentialing. *Principles of addictions and the law: Applications in forensic, mental health, and medical practice*. 2010;(American College of Legal Medicine. (2007), Peer review. Legal Medicine Perspectives, 16(4), 68-69.American College of Legal Medicine. (2008). Physician references. Legal Medicine Q & A, 7(3), 1-2.American College of Medical Quality (ACMQ). (2008a), Profe):55-74. doi:https://dx.doi.org/10.1016/B978-0-12-496736-6.00004-9

180. Grogan K. Recovering voices from the margins: How female physicians experience issues of addiction and impairment. *Dissertation Abstracts International Section A: Humanities and Social Sciences*. 2013;73(12-A(E)):No-Specified.

181. Guidera A, Field R, Love RL. Re: who will have the 'guts' to quantify the decline in surgical skills of older surgeons? A commentary on the paper that shows that this occurs in thyroid surgeons. *Clinical otolaryngology : official journal of ENT-UK ; official journal of Netherlands Society for Oto-Rhino-Laryngology & Cervico-Facial Surgery*. 2012;37(5):425-7. doi:https://dx.doi.org/10.1111/coa.12020

182. Gunderman RB, Grogan K. Physician impairment and professionalism. *AJR American journal of roentgenology*. 2012;199(5):W543-4. doi:https://dx.doi.org/10.2214/AJR.11.8146

183. Gundersen DC. Impaired psychiatrists. *Malpractice and liability in psychiatry*. 2022;(AMA Council on Mental Health (1973). The sick physician. Impairment by psychiatric disorders, including alcoholism and drug dependence. JAMA, 223(6), 684-687 https://pubmed.ncbi.nlm.nih.gov/4739202 https://dx.doi.org/10.1001/jama.1973.03220060058020Americ):289-295. doi:https://dx.doi.org/10.1007/978-3-030-91975-7_36

184. Guraya SS, Menezes P, Lawrence IN, Guraya SY, Rashid-Doubell F. Evaluating the impact of COVID-19 pandemic on the physicians' psychological health: A systematic scoping review. *Frontiers in medicine*. 2023;10(101648047):1071537. doi:https://dx.doi.org/10.3389/fmed.2023.1071537

185. Haddad T. Cognitive assessment in the practice of medicine--dealing with the aging physician. *Physician executive*. 2013;39(4):14-20.

186. Hagan JC, 3rd. When good docs go bad: the impaired physician. *Missouri medicine*. 2002;99(4):153-4.

187. Hall PB. "What is a physician health program?" The West Virginia Medical Professionals Health Program mission. *The West Virginia medical journal*. 2007;103(5):32-34.

188. Halperin EC, Andolsek KM, Jackson GW, Weinerth J. Pre-placement screening of resident physicians by substance abuse testing: Efficacy, cost, and physician opinions. *Drugs: Education, Prevention & Policy*. 2008;15(1):77-91. doi:https://dx.doi.org/10.1080/09687630701267358

189. Harbison KG. New law avoids unnecessary punishment of impaired physicians. *Minnesota medicine*. 1994;77(9):41-4.

190. Harmon L, Pomm RM. Evaluation, treatment, and monitoring of disruptive physician behavior. *Psychiatric Annals*. 2004;34(10):770-774. doi:https://dx.doi.org/10.3928/0048-5713-20041001-16

191. Harrison J. Doctors' health and fitness to practise: the need for a bespoke model of assessment. *Occupational medicine (Oxford, England)*. 2008;58(5):323-7. doi:https://dx.doi.org/10.1093/occmed/kqn079

192. Harrison J. Doctors' health and fitness to practise: assessment models. *Occupational medicine (Oxford, England)*. 2008;58(5):318-22. doi:https://dx.doi.org/10.1093/occmed/kqn078

193. Harty-Golder B. Reporting impaired pathologist. *MLO: medical laboratory observer*. 2009;41(2):36.

194. Hassan TM, Sikander S, Mazhar N, Munshi T, Galbraith N, Groll D. Canadian psychiatrists' attitudes to becoming mentally ill. *British Journal of Medical Practitioners*. 2013;6(3):a619.

195. Hatcher S. How doctors become patients. *First do no self-harm: Understanding and promoting physician stress resilience*. 2013;(aan het Rot, M., Mathew, S. J., & Charney, D. S. (2009). Neurobiological mechanisms in major depressive disorder. Canadian Medical Association Journal, 180(3), 305-313. 2009-05181-007. https://dx.doi.org/10.1503/cmaj.080697 https://pubmed.ncbi.nlm.nih.gov):171-192.

196. Hatton KW, Bacon JD, McKinney K, Schell RM. A Novel Tool to Guide Reintegration of Anesthesiologists Into Clinical and Academic Work After Concussion. *A&A practice*. 2019;12(9):336-339. doi:https://dx.doi.org/10.1213/XAA.0000000000000957

197. Heymann WR. Assessing the Competence of Aging Physicians Who Are Young at Heart. *JAMA dermatology*. 2018;154(8):875-876. doi:https://dx.doi.org/10.1001/jamadermatol.2018.1371

198. Hickson GB, Peabody T, Hopkinson WJ, Reiter CE, 3rd. Cognitive Skills Assessment for the Aging Orthopaedic Surgeon: AOA Critical Issues. *The Journal of bone and joint surgery American volume*. 2019;101(2):e7. doi:https://dx.doi.org/10.2106/JBJS.18.00470

199. Higgins MCSS, Siddiqui AA, Kosowsky T, et al. Burnout, Professional Fulfillment, Intention to Leave, and Sleep-Related Impairment among Radiology Trainees across the United States (US): A Multisite Epidemiologic Study. *Academic Radiology*. 2022;29(Supplement 5):S118-S125. doi:https://dx.doi.org/10.1016/j.acra.2022.01.022

200. Ho JD, Kuo NW, Tsai CY, Liou SW, Lin HC. Surgeon age and operative outcomes for primary rhegmatogenous retinal detachment: a 3-year nationwide population-based study. *Eye (London, England)*. 2010;24(2):290-6. doi:https://dx.doi.org/10.1038/eye.2009.99

201. Hobbs T. Addressing perceptions of the impaired physician. *Pennsylvania medicine*. 1998;101(2):11.

202. Holtman MC. Disciplinary careers of drug-impaired physicians. *Social science & medicine (1982)*. 2007;64(3):543-53.

203. Homan RV. Commentary on "The aging physician and surgeon" by Sataloff et al. *Ear, Nose and Throat Journal*. 2016;95(4-5):130-132.

204. Hotchkiss N, Early S. The differences in keeping both male and female physicians healthy. *The health care manager*. 2009;28(4):299-310. doi:https://dx.doi.org/10.1097/HCM.0b013e3181bdece1

205. Hotchkiss NF. Predictors of reactivation and gender differences at a physician health program by age, marital status, primary presenting problem, referral source, and referral status. *Dissertation Abstracts International: Section B: The Sciences and Engineering*. 2008;69(2-B):1369.

206. Huizinga CRH, de Kam ML, Stockmann HBAC, van Gerven JMA, Cohen AF, van der Bogt KEA. Evaluating Fitness to Perform in Surgical Residents after Night Shifts and Alcohol Intoxication: The development of a "Fit-to-Perform" test. *Journal of surgical education*. 2018;75(4):968-977. doi:https://dx.doi.org/10.1016/j.jsurg.2018.01.010

207. Hulse G, Sim MG, Khong E. Management of the impaired doctor. *Australian family physician*. 2004;33(9):703-7.

208. Hulse GK, O'Neil G, Arnold-Reed DE. Management of an opioid-impaired anaesthetist by implantable naltrexone. *Journal of Substance Use*. 2004;9(2):86-90. doi:https://dx.doi.org/10.1080/14659890410001665087

209. Hulse GK, O'Neil G, Hatton M, Paech MJ. Use of oral and implantable naltrexone in the management of the opioid impaired physician. *Anaesthesia and intensive care*. 2003;31(2):196-201.

210. Ilse R. Leadership ethics and the challenge of the aging physician. *Healthcare Management Forum*. 2013;26(3):166-168. doi:https://dx.doi.org/10.1016/j.hcmf.2013.08.001

211. Jennings ML. Medical student burnout: Interdisciplinary exploration and analysis. *Journal of Medical Humanities*. 2009;30(4):253-269. doi:https://dx.doi.org/10.1007/s10912-009-9093-5

212. Johnson BA. Dealing with the impaired physician. *American family physician*. 2009;80(9):1007.

213. Johnson RS, Fowler JC, Sikes KA, Allen JG, Oldham JM. Treatment of Depression in Voluntary Versus Mandated Physicians. *The journal of the American Academy of Psychiatry and the Law*. 2015;43(4):476-82.

214. Jones JW, McCullough LB. The question of an impaired surgeon dilemma. *Journal of vascular surgery*. 2012;56(6):1761-2. doi:https://dx.doi.org/10.1016/j.jvs.2012.10.063

215. Jones JW, McCullough LB, Richman BW. An impaired surgeon, a conflict of interest, and supervisory responsibilities. *Surgery*. 2004;135(4):449-51.

216. Jullian B, Deltour M, Franchitto N. The consumption of psychoactive substances among French physicians: how do they perceive the creation of a dedicated healthcare system? *Frontiers in psychiatry*. 2023;14(101545006):1249434. doi:https://dx.doi.org/10.3389/fpsyt.2023.1249434

217. Kast KA, Avery J. The case of Dr. Sigi Halsted: Overdose in the OR. *Early career physician mental health and wellness: A clinical casebook*. 2019;(Boyd, J. W., & Knight, J. R. (2015). Substance use disorders among physicians. In: GalanterM, KleberHD, BradyKT, editors. The American Psychiatric Publishing Textbook of substance abuse treatment, 5th Edition. American Psychiatric Publishing. 2015. https:):73-86. doi:https://dx.doi.org/10.1007/978-3-030-10952-3_7

218. Kataria N, Brown N, McAvoy P, Majeed A, Rhodes M. A retrospective study of cognitive function in doctors and dentists with suspected performance problems: an unsuspected but significant concern. *JRSM open*. 2014;5(5):2042533313517687. doi:https://dx.doi.org/10.1177/2042533313517687

219. Katlic MR, Coleman J. The aging surgeon. *Annals of surgery*. 2014;260(2):199-201. doi:https://dx.doi.org/10.1097/SLA.0000000000000667

220. Katlic MR, Coleman J. The Aging Surgeon. *Advances in surgery*. 2016;50(1):93-103. doi:https://dx.doi.org/10.1016/j.yasu.2016.03.008

221. Katlic MR, Coleman J, Russell MM. Assessing the Performance of Aging Surgeons. *JAMA*. 2019;321(5):449-450. doi:https://dx.doi.org/10.1001/jama.2018.22216

222. Katz JD. Challenges for the aging physician. *Connecticut medicine*. 2002;66(9):539-542.

223. Katz JD. The aging anesthesiologist. *Current Opinion in Anaesthesiology*. 2016;29(2):206-211. doi:https://dx.doi.org/10.1097/ACO.0000000000000299

224. Katz JD. The impaired and/or disabled anesthesiologist. *Current Opinion in Anaesthesiology*. 2017;30(2):217-222. doi:https://dx.doi.org/10.1097/ACO.0000000000000423

225. Kay M, Del Mar CB, Mitchell G. Does legislation reduce harm to doctors who prescribe for themselves? *Australian family physician*. 2005;34(1-2):94-6.

226. Kels CG. "Cognitive testing of older clinicians prior to recredentialing": Comment. *JAMA: Journal of the American Medical Association*. 2020;323(19):1974-1975. doi:https://dx.doi.org/10.1001/jama.2020.4260

227. Kevern T. *How Do Depression, Sleep Impairment, and Burnout Impact Medical Residents’ and Fellows’ Medical Errors? An Examination Using Structural Equation Modeling*. 2023.

228. Khong E, Sim MG, Hulse G. The identification and management of the drug impaired doctor. *Australian family physician*. 2002;31(12):1097-100.

229. Killewich LA. The impaired surgeon: revisiting Halstead. *Journal of vascular surgery*. 2009;50(2):440-1. doi:https://dx.doi.org/10.1016/j.jvs.2009.05.001

230. Kimak A, Wozniacka A. How old is too old to work for physicians? *Postepy dermatologii i alergologii*. 2023;40(3):368-371. doi:https://dx.doi.org/10.5114/ada.2023.128977

231. Klinglesmith RE. A formative evaluation of the Kentucky Physicians Health Foundation Impaired Physicians Program. *Dissertation Abstracts International: Section B: The Sciences and Engineering*. 2000;61(2-B):1086.

232. Knight JR. A 35-year-old physician with opioid dependence: Reply. *JAMA: Journal of the American Medical Association*. 2005;293(3):294. doi:https://dx.doi.org/10.1001/jama.293.3.294-c

233. Knight JR, Sanchez LT, Sherritt L, Bresnahan LR, Fromson JA. Outcomes of a monitoring program for physicians with mental and behavioral health problems. *Journal of Psychiatric Practice*. 2007;13(1):25-32. doi:https://dx.doi.org/10.1097/00131746-200701000-00004

234. Knight JR, Sanchez LT, Sherritt L, Bresnahan LR, Silveria JM, Fromson JA. Monitoring physician drug problems: Attitudes of participants. *Journal of Addictive Diseases*. 2002;21(4):27-36. doi:https://dx.doi.org/10.1300/J069v21n04_03

235. Knoepflmacher D. The case of Erik Quimby: A disruptive physician in training. *Early career physician mental health and wellness: A clinical casebook*. 2019;(Accreditation Council for Graduate Medical Education. (2013). ACGME common program requirements. 2013.Alert, S. E. (2008). Behaviors that undermine a culture of safety. Sentinel Event Alert. 2008(40):1-3.American Psychiatric Association. (2013). Diagnosti):87-100. doi:https://dx.doi.org/10.1007/978-3-030-10952-3_8

236. Korr M. RI's Physician Health Program: Serving practitioners for more than four decades. *Rhode Island medical journal (2013)*. 2023;106(10):66-68.

237. Krebs-Markrich J, Perrine KW. Defending the impaired physician. *Virginia medical quarterly : VMQ*. 1996;123(4 Suppl):14-6.

238. Kwah J, Weintraub J, Fallar R, Ripp J. The Effect of Burnout on Medical Errors and Professionalism in First-Year Internal Medicine Residents. *Journal of graduate medical education*. 2016;8(4):597-600.

239. La Hee F. The impaired physician. *The West Indian medical journal*. 2005;54(2):95-6.

240. Ladouceur R. Should older family physicians retire? *Canadian family physician Medecin de famille canadien*. 2012;58(1):11.

241. Lalloo D, Ghafur I, Macdonald EB. Doctor and dentist contacts with an NHS occupational health service. *Occupational medicine (Oxford, England)*. 2013;63(4):291-3. doi:https://dx.doi.org/10.1093/occmed/kqt029

242. Langevin R, Glancy GD, Curnoe S, Bain J. Physicians who commit sexual offenses: Are they different from other sex offenders? *The Canadian Journal of Psychiatry / La Revue canadienne de psychiatrie*. 1999;44(8):775-786.

243. Lawson ND. Who Benefits from Physician Wellness? *Canadian journal of psychiatry Revue canadienne de psychiatrie*. 2020;65(1):61-62. doi:https://dx.doi.org/10.1177/0706743719890729

244. Lawson ND, Boyd JW. Do state physician health programs encourage referrals that violate the Americans with Disabilities Act? *International journal of law and psychiatry*. 2018;56(grp, 7806862):65-70. doi:https://dx.doi.org/10.1016/j.ijlp.2017.12.004

245. Lawson ND, Boyd JW. Flaws in the methods and reporting of physician health program outcome studies. *General hospital psychiatry*. 2018;54(fnk, 7905527):65-66. doi:https://dx.doi.org/10.1016/j.genhosppsych.2018.06.002

246. Lee HK, Bluestone H. Ethical and forensic considerations in substance abuse treatment. *Psychiatric-legal decision making by the mental health practitioner: The clinician as de facto magistrate*. 1994:187-201.

247. Lee L, Weston W. The aging physician. *Canadian family physician Medecin de famille canadien*. 2012;58(1):17-8.

248. Liang BA, Connelly CbNR, Raghunathan K. To tell the truth: potential liability for concealing physician impairment. *Journal of Clinical Anesthesia*. 2007;19(8):638-641. doi:https://dx.doi.org/10.1016/j.jclinane.2007.10.001

249. LoboPrabhu SM, Molinari VA, Hamilton JD, Lomax JW. The aging physician with cognitive impairment: approaches to oversight, prevention, and remediation. *The American journal of geriatric psychiatry : official journal of the American Association for Geriatric Psychiatry*. 2009;17(6):445-54. doi:https://dx.doi.org/10.1097/JGP.0b013e31819e2d7e

250. Loevenich A, Schmidt R, Schifferdecker M. Doctors as patients - The problem of the mentally ill physician. *Fortschritte der Neurologie Psychiatrie*. 1996;64(9):344-352. doi:https://dx.doi.org/10.1055/s-2007-996576

251. Love L, McDowelle D. Developing a comprehensive wellness program for medical students. *Exploring the pressures of medical education from a mental health and wellness perspective*. 2018;(Brazeau, C. M. (2010). The Surest Way to Prevent Burnout in Medical School and Beyond. Academic Medicine, 85(4), 569-570 2010-07724-001. https://dx.doi.org/10.1097/ACM.0b013e3181d2d643Dyrbye, L. N., West, C. P., Satele, D., Boone, S., Tan, L., Sloan, J.):190-208. doi:https://dx.doi.org/10.4018/978-1-5225-2811-1.ch008

252. Lusilla P. Psychiatrists treating doctors. *European Psychiatry*. 2012;27(SUPPL. 1)doi:https://dx.doi.org/10.1016/S0924-9338%2812%2974132-9

253. Lusilla P, Gual A, Roncero C, et al. Dual diagnosis in inpatient physicians: Prevalence and clinical characteristics. *Mental Health and Substance Use: dual diagnosis*. 2008;1(1):10-20. doi:https://dx.doi.org/10.1080/17523280701724189

254. Mache S, Baresi L, Bernburg M, Vitzthum K, Groneberg D. Being prepared to work in Gynecology Medicine: evaluation of an intervention to promote junior gynecologists professionalism, mental health and job satisfaction. *Archives of gynecology and obstetrics*. 2017;295(1):153-162. doi:https://dx.doi.org/10.1007/s00404-016-4223-6

255. Magnavita N. Management of impaired physicians in Europe. *La Medicina del lavoro*. 2006;97(6):762-73.

256. Magnavita N, Magnavita G, Bergamaschi A. The impaired radiologist. *La Radiologia medica*. 2010;115(5):826-38. doi:https://dx.doi.org/10.1007/s11547-010-0539-7

257. Mandell WJ. An approach to the impaired physician. *Physician executive*. 1994;20(5):7-14.

258. Mansky P, Wang C, Morse LJ. An Impaired Physician's Physician. *The virtual mentor : VM*. 2003;5(9)doi:https://dx.doi.org/10.1001/virtualmentor.2003.5.9.ccas1-0309

259. Mansky PA. Physician health programs and the potentially impaired physician with a substance use disorder. *Psychiatric services (Washington, DC)*. 1996;47(5):465-7.

260. Mareiniss DP. Decreasing GME training stress to foster residents' professionalism. *Academic medicine : journal of the Association of American Medical Colleges*. 2004;79(9):825-31.

261. Markar SR, Mackenzie H, Lagergren P, Lagergren J. Surgeon Age in Relation to Prognosis After Esophageal Cancer Resection. *Annals of surgery*. 2018;268(1):100-105. doi:https://dx.doi.org/10.1097/SLA.0000000000002260

262. Marshall EJ. Doctors' health and fitness to practise: treating addicted doctors. *Occupational medicine (Oxford, England)*. 2008;58(5):334-40. doi:https://dx.doi.org/10.1093/occmed/kqn081

263. Masri R, Kadhum M, Farrell SM, Khamees Aa, Al-Taiar H, Molodynski A. Wellbeing and mental health amongst medical students in Jordan: a descriptive study. *International review of psychiatry (Abingdon, England)*. 2019;31(7-8):619-625. doi:https://dx.doi.org/10.1080/09540261.2019.1670402

264. Mattingly DE, Curtis LG. Physician assistant impairment. A peer review program for North Carolina. *North Carolina medical journal*. 1996;57(4):233-5.

265. Maves MD. Chapter 7: The impaired physician. *Otolaryngology - Head and Neck Surgery*. 1996;115(3):213-219. doi:http://dx.doi.org/10.1016/S0194-5998(96)70030-X

266. McGovern MP, Angres DH, Leon S. Differential therapeutics and the impaired physician: patient-treatment matching by specificity and intensity. *Journal of addictive diseases*. 1998;17(2):93-107.

267. McGovern MP, Angres DH, Leon S. Characteristics of physicians presenting for assessment at a behavioral health center. *Journal of addictive diseases*. 2000;19(2):59-73.

268. McIntyre BW, Hamolsky MW. The impaired physician and the role of the Board of Medical Licensure and Discipline. *Rhode Island medicine*. 1994;77(10):357-8.

269. McKenna M. Aging gracefully?: patient safety advocates call for ongoing skills assessments for older physicians. *Annals of emergency medicine*. 2011;58(3):A15-7.

270. McLellan AT, Skipper GS, Campbell M, DuPont RL. Five year outcomes in a cohort study of physicians treated for substance use disorders in the United States. *BMJ (Clinical research ed)*. 2008;337(8900488, bmj, 101090866):a2038. doi:https://dx.doi.org/10.1136/bmj.a2038

271. McNamara RM, Margulies JL. Chemical dependency in emergency medicine residency programs: perspective of the program directors. *Annals of emergency medicine*. 1994;23(5):1072-6.

272. McTaggart LS, Walker JP. The relationship between resident physician burnout and its' effects on patient care, professionalism, and academic achievement: A review of the literature. *Health Sciences Review*. 2022;4((McTaggart, Walker) Department of Surgery, University of Texas Medical Branch, 301 University Blvd. 0353, Galveston, TX 77555, United States):100049. doi:https://dx.doi.org/10.1016/j.hsr.2022.100049

273. Meeks LM, Cleary J, Horwitz A, et al. Analysis of Depressive Symptoms and Perceived Impairment Among Physicians Across Intern Year. *JAMA network open*. 2022;5(1):e2144919. doi:https://dx.doi.org/10.1001/jamanetworkopen.2021.44919

274. Merlo LJ, Altenburger KM, Gold MS. Physicians' experiences with impaired colleagues [1]. *JAMA*. 2010;304(17):1895. doi:https://dx.doi.org/10.1001/jama.2010.1557

275. Merlo LJ, Campbell MD, Shea C, et al. Essential components of physician health program monitoring for substance use disorder: A survey of participants 5 years post successful program completion. *The American journal on addictions*. 2022;31(2):115-122. doi:https://dx.doi.org/10.1111/ajad.13257

276. Merlo LJ, Campbell MD, Skipper GE, Shea CL, DuPont RL. Outcomes for Physicians With Opioid Dependence Treated Without Agonist Pharmacotherapy in Physician Health Programs. *Journal of substance abuse treatment*. 2016;64(kai, 8500909):47-54. doi:https://dx.doi.org/10.1016/j.jsat.2016.02.004

277. Merlo LJ, Gold MS. Prescription opioid abuse and dependence among physicians: hypotheses and treatment. *Harvard review of psychiatry*. 2008;16(3):181-94. doi:https://dx.doi.org/10.1080/10673220802160316

278. Merlo LJ, Greene WM. Physician views regarding substance use-related participation in a state physician health program. *The American journal on addictions*. 2010;19(6):529-33. doi:https://dx.doi.org/10.1111/j.1521-0391.2010.00088.x

279. Merlo LJ, Polles A, Sutton JA, Greene WM, Thompson KM. Substance use disorders and recovery. *Understanding and cultivating well-being for the pediatrician: A compilation of the latest evidence in pediatrician well-being science*. 2023:99-116. doi:https://dx.doi.org/10.1007/978-3-031-10843-3_5

280. Merlo LJ, Singhakant S, Cummings SM, Cottler LB. Reasons for misuse of prescription medication among physicians undergoing monitoring by a physician health program. *Journal of addiction medicine*. 2013;7(5):349-53. doi:https://dx.doi.org/10.1097/ADM.0b013e31829da074

281. Micko A, Knopp K, Knosp E, Wolfsberger S. Microsurgical Performance After Sleep Interruption: A NeuroTouch Simulator Study. *World neurosurgery*. 2017;106(101528275):92-101. doi:https://dx.doi.org/10.1016/j.wneu.2017.06.142

282. Miles SH. A challenge to licensing boards: The stigma of mental illness. *JAMA: Journal of the American Medical Association*. 1998;280(10):865. doi:https://dx.doi.org/10.1001/jama.280.10.865

283. Miller L. Doctors, their mental health and capacity for work. *Occupational Medicine*. 2009;59(1):53-55. doi:https://dx.doi.org/10.1093/occmed/kqn111

284. Moon MR. Early- and late-career surgeon deficiencies in complex cases. *The Journal of thoracic and cardiovascular surgery*. 2022;164(3):1023-1025. doi:https://dx.doi.org/10.1016/j.jtcvs.2021.11.080

285. Moskowitz PS. Re: "Beyond substance abuse: stress, burnout, and depression as causes of physician impairment and disruptive behavior". *Journal of the American College of Radiology : JACR*. 2010;7(4):313-5. doi:https://dx.doi.org/10.1016/j.jacr.2010.01.008

286. Mossman D, Farrell HM. Physician impairment: When should you report? *Current Psychiatry*. 2011;10(9):67-71.

287. Moulton D. Older doctors under increasing regulatory scrutiny. *CMAJ : Canadian Medical Association journal = journal de l'Association medicale canadienne*. 2016;188(12):E270. doi:https://dx.doi.org/10.1503/cmaj.109-5298

288. Mull CC, Thompson AD, Rappaport DI, Gartner JC, Jr., Bowman WR. A Call to Restore Your Calling: Self-Care of the Emergency Physician in the Face of Life-Changing Stress-Part 3 of 6: Physician Illness and Impairment. *Pediatric emergency care*. 2019;35(8):585-588. doi:https://dx.doi.org/10.1097/PEC.0000000000001896

289. Myers MF. Physician impairment: is it relevant to academic psychiatry? *Academic psychiatry : the journal of the American Association of Directors of Psychiatric Residency Training and the Association for Academic Psychiatry*. 2008;32(1):39-43. doi:https://dx.doi.org/10.1176/appi.ap.32.1.39

290. Nelson HD, Matthews AM, Girard DE, Bloom JD. Substance-impaired physicians probationary and voluntary treatment programs compared. *The Western journal of medicine*. 1996;165(1-2):31-6.

291. O'Connor M. Alcohol Consumption and Impairment of Surgeons: A Case for Total Abstinence? *Journal of law and medicine*. 2017;24(3):556-64.

292. O'Connor PG, Spickard A, Jr. Physician impairment by substance abuse. *The Medical clinics of North America*. 1997;81(4):1037-52.

293. Ohlsen JD. The board's role with impaired physicians. *Trustee : the journal for hospital governing boards*. 2006;59(6):32-3.

294. Palhares-Alves HN, Vieira DL, Laranjeira RR, Vieira JE, Nogueira-Martins LA. Clinical and demographic profile of anesthesiologists using alcohol and other drugs under treatment in a pioneering program in Brazil. *Revista brasileira de anestesiologia*. 2012;62(3):356-64. doi:https://dx.doi.org/10.1016/S0034-7094(12)70136-8

295. Pandya SK. At what age should surgeons be forced to stop operating in private hospitals? *The National medical journal of India*. 2007;20(2):93-4.

296. Paris RT, Canavan DI. Physician substance abuse impairment: anesthesiologists vs. other specialties. *Journal of addictive diseases*. 1999;18(1):1-7.

297. Parry AL, Brooks E, Early SR. A Retrospective Cross-Sectional Review of Resident Care-Seeking at a Physician Health Program. *Academic psychiatry : the journal of the American Association of Directors of Psychiatric Residency Training and the Association for Academic Psychiatry*. 2018;42(5):636-641. doi:https://dx.doi.org/10.1007/s40596-018-0917-3

298. Peisah C, Adler RG, Williams BW. Australian pathways and solutions for dealing with older impaired doctors: a prevention model. *Internal medicine journal*. 2007;37(12):826-31.

299. Peisah C, Wilhelm K. The impaired ageing doctor. *Internal medicine journal*. 2002;32(9-10):457-9.

300. Peisah C, Wilhelm K. Physician don't heal thyself: a descriptive study of impaired older doctors. *International psychogeriatrics*. 2007;19(5):974-84.

301. Pendergast W, Scarborough J. Physician health vs. impairment: the North Carolina Physicians Health Program. *North Carolina medical journal*. 2009;70(1):59-61.

302. Penneau M. Expertise of article R. 4124-3 of the Public Health Code: Suspension of activity due to a pathological condition. *Annales Medico-Psychologiques*. 2007;165(1):63-65. doi:https://dx.doi.org/10.1016/j.amp.2006.09.010

303. Petersen-Crair P, Marangell L, Flack J, Harper R, Soety E, Gabbard GO. An impaired physician with complex comorbidity. *The American journal of psychiatry*. 2003;160(5):850-4.

304. Pham JC, Pronovost PJ, Skipper GE. Identification of physician impairment. *JAMA*. 2013;309(20):2101-2. doi:https://dx.doi.org/10.1001/jama.2013.4635

305. Pham JC, Pronovost PJ, Skipper GE. "Protection of patients from physician substance misuse": In reply. *JAMA: Journal of the American Medical Association*. 2013;310(13):1403-1404. doi:https://dx.doi.org/10.1001/jama.2013.277978

306. Pinzur MS. The Aging Surgeon. *Foot & ankle international*. 2018;39(1):129. doi:https://dx.doi.org/10.1177/1071100717732774

307. Pitkanen M, Hurn J, Kopelman MD. Doctors' health and fitness to practise: performance problems in doctors and cognitive impairments. *Occupational medicine (Oxford, England)*. 2008;58(5):328-33. doi:https://dx.doi.org/10.1093/occmed/kqn080

308. Polles A, Bundy C, Jacobs W, Merlo LJ. Adaptations to substance use disorder monitoring by physician health programs in response to COVID-19. *Journal of substance abuse treatment*. 2021;125(kai, 8500909):108281. doi:https://dx.doi.org/10.1016/j.jsat.2021.108281

309. Polles AG, Jacobs WS, Brazle C, Merlo LJ. The Role of Alcohol Biomarkers in Detecting a Physician's COVID-19-Related Acute Stress Response: A Case Report. *Journal of Addiction Medicine*. 2022;16(1):E62-E65. doi:https://dx.doi.org/10.1097/ADM.0000000000000865

310. Polles AG, Williams MK, Phalin BR, Teitelbaum S, Merlo LJ. Neuropsychological impairment associated with substance use by physicians. *Journal of the neurological sciences*. 2020;411(jbj, 0375403):116714. doi:https://dx.doi.org/10.1016/j.jns.2020.116714

311. Poma PA. Wellness committees address physician impairment. *Bulletin of the American College of Surgeons*. 2000;85(2):20-5.

312. Pomm RM, Harmon L. Evaluation and posttreatment monitoring of the impaired physician. *Psychiatric Annals*. 2004;34(10):786-789. doi:https://dx.doi.org/10.3928/0048-5713-20041001-19

313. Posen S. The portrayal of the doctor in non-medical literature: the impaired doctor. *The Medical journal of Australia*. 1997;166(1):48-51.

314. Powell T. OK, Boomer, MD: The Rights of Aging Physicians and the Health of Our Communities. *The Hastings Center report*. 2020;50(6):3. doi:https://dx.doi.org/10.1002/hast.1191

315. Price M, Meyer DJ. Fitness-for-duty evaluations of physicians and health care professionals: Treating providers and protecting the public. *Clinical guide to mental disability evaluations*. 2013;(Alexander, B. H., Checkoway, H., Nagahama, S. I., et al. (2000). Cause-specific mortality risks of anesthesiologists. Anesthesiology 93, 922-930 (2000)American Board of Medical Specialties.Board Certification Editorial Background, http://www.abms,org/news):337-367. doi:https://dx.doi.org/10.1007/978-1-4614-5447-2_13

316. Price M, Meyer DJ. The aging physician and other professionals assessing fitness for duty. *Geriatric forensic psychiatry: Principles and practice*. 2018;(Age Discrimination Act of 1975, amended, Pub.L. 95-478, 92 Stat 1555 (1978).Age Discrimination in Employment Act of 1967(ADEA), Pub.L. 114-138 (1967).American Medical Association. (2016). Code of Medical Ethics. Chapter 9. Opinion on professional rights a):321-328.

317. Raje D. The impaired physician. *The West Indian medical journal*. 1995;44(2):39.

318. Raskin MM. Of a certain age: When are radiologists too old to practice? *Applied Radiology*. 2018;47(11):6-7.

319. Reade JM. Impaired Physicians: Role of the Occupational Psychiatrist. *Psychiatric Annals*. 2006;36(11):799-803.

320. Reich J, Kelly M. Empirical findings of fitness-for-duty evaluations. *MedEdPublish (2016)*. 2018;7(9918418288706676):258. doi:https://dx.doi.org/10.15694/mep.2018.0000258.1

321. Reid WH. Evaluating and Treating Disabled or Impaired Colleagues. *Journal of Psychiatric Practice*. 2007;13(1):44-48. doi:https://dx.doi.org/10.1097/00131746-200701000-00007

322. Reyes AJ, Ramcharan K, Sharma S. Multiple sclerosis in a postgraduate student of anaesthesia: illness in doctors and fitness to practice. *BMJ case reports*. 2016;2016(101526291)doi:https://dx.doi.org/10.1136/bcr-2015-213845

323. Roberts LW, Warner TD, Rogers M, Horwitz R, Redgrave G. Medical student illness and impairment: a vignette-based survey study involving 955 students at 9 medical schools. *Comprehensive psychiatry*. 2005;46(3):229-37.

324. Robinson DBT, James OP, Hopkins L, et al. Stress and Burnout in Training; Requiem for the Surgical Dream. *Journal of surgical education*. 2020;77(1):e1-e8. doi:https://dx.doi.org/10.1016/j.jsurg.2019.07.002

325. Rose GL, Brown RE, Jr. The impaired anesthesiologist: not just about drugs and alcohol anymore. *Journal of clinical anesthesia*. 2010;22(5):379-84. doi:https://dx.doi.org/10.1016/j.jclinane.2009.09.009

326. Rose J, Campbell M, Skipper G. Emergency physicians with substance abuse: Prognosis for recovery a 5-year outcome study. *Western Journal of Emergency Medicine*. 2014;15(1)doi:https://dx.doi.org/10.5811/westjem.2013.7.17871

327. Rose JS, Campbell MD, Yellowlees P, Skipper GE, DuPont RL. Family Medicine Physicians With Substance Use Disorder: A 5-year Outcome Study. *Journal of addiction medicine*. 2017;11(2):93-97. doi:https://dx.doi.org/10.1097/ADM.0000000000000278

328. Rosenstein AH. Hospital administration response to physician stress and burnout. *Hospital practice (1995)*. 2019;47(5):217-220. doi:https://dx.doi.org/10.1080/21548331.2019.1688596

329. Ross S. Identifying an impaired physician. *The virtual mentor : VM*. 2003;5(12)doi:https://dx.doi.org/10.1001/virtualmentor.2003.5.12.cprl1-0312

330. Sadavoy J. The aging physician. *Journal of Geriatric Psychiatry*. 1994;27(2):265-279.

331. Saddawi-Konefka D, Brown A, Eisenhart I, Hicks K, Barrett E, Gold JA. Consistency Between State Medical License Applications and Recommendations Regarding Physician Mental Health. *JAMA*. 2021;325(19):2017-2018. doi:https://dx.doi.org/10.1001/jama.2021.2275

332. Saenger R. The case of Ramona Williams: Losing touch. *Early career physician mental health and wellness: A clinical casebook*. 2019;(Kirkbride, J. B., et al. (2012). Incidence of schizophrenia and other psychoses in England, 1950-2009: a systematic review and meta-analyses. PLoS One. 2012;7(3):e31660. 2012-09797-001. https://dx.doi.org/10.1371/journal.pone.0031660 https://pubmed.ncbi.n):35-43. doi:https://dx.doi.org/10.1007/978-3-030-10952-3_4

333. Sagalyn A. The aging physician: when practice doesn't make perfect. *Maryland medicine : MM : a publication of MEDCHI, the Maryland State Medical Society*. 2013;14(3):13-5.

334. Samenow CP. Physician health, impairment, and misconduct. *The behavioral sciences and health care, 3rd ed*. 2012:308-314.

335. Sansone RA, Wiederman MW, Sansone LA. Physician mental health and substance abuse. What are state medical licensure applications asking? *Archives of family medicine*. 1999;8(5):448-51.

336. Sataloff RT, Hawkshaw M, Kutinsky J, Maitz EA. The aging physician and surgeon. *Ear, nose, & throat journal*. 2016;95(4-5):E35-48.

337. Saunders D. Substance abuse and dependence in anaesthetists. *Best practice & research Clinical anaesthesiology*. 2006;20(4):637-43.

338. Saunders D. The older anaesthetist. *Best practice & research Clinical anaesthesiology*. 2006;20(4):645-51.

339. Saver JL. Best Practices in Assessing Aging Physicians for Professional Competency. *JAMA*. 2020;323(2):127-129. doi:https://dx.doi.org/10.1001/jama.2019.20249

340. Schenarts PJ, Cemaj S. The Aging Surgeon: Implications for the Workforce, the Surgeon, and the Patient. *The Surgical clinics of North America*. 2016;96(1):129-38. doi:https://dx.doi.org/10.1016/j.suc.2015.09.009

341. Schouten R. Impaired physicians: is there a duty to report to state licensing boards? *Harvard review of psychiatry*. 2000;8(1):36-9.

342. Schwartz RP, White RK, McDuff DR, Johnson JL. Four years experience of a hospital's impaired physician committee. *Journal of addictive diseases*. 1995;14(2):13-21.

343. Sheikh MH, Waqas A, Naveed S, et al. Association of cognitive impairment with sleeping difficulties, anxiety and depression among Pakistani physicians. *JPMA The Journal of the Pakistan Medical Association*. 2018;68(6):932-935.

344. Sherertz RJ, Karchmer TB. Surgical site infection as a surrogate marker of physician impairment. *Infection control and hospital epidemiology*. 2009;30(11):1120-2. doi:https://dx.doi.org/10.1086/647982

345. Sherwood R, Bismark M. The ageing surgeon: a qualitative study of expert opinions on assuring performance and supporting safe career transitions among older surgeons. *BMJ quality & safety*. 2020;29(2):113-121. doi:https://dx.doi.org/10.1136/bmjqs-2019-009596

346. Shilnikova N, Momoli F, Taher MK, et al. Should we screen aging physicians for cognitive decline? *Aging & mental health*. 2023;(d46, 9705773):1-12. doi:https://dx.doi.org/10.1080/13607863.2023.2252371

347. Shore JH. Progress in treatments for substance-impaired physicians. *The Western journal of medicine*. 1996;165(1-2):81-2.

348. Sinskey JL, Margolis RD, Vinson AE. The Wicked Problem of Physician Well-Being. *Anesthesiology clinics*. 2022;40(2):213-223. doi:https://dx.doi.org/10.1016/j.anclin.2022.01.001

349. Siu LW, Boet S, Borges BCR, et al. High-fidelity simulation demonstrates the influence of anesthesiologists' age and years from residency on emergency cricothyroidotomy skills. *Anesthesia and analgesia*. 2010;111(4):955-60. doi:https://dx.doi.org/10.1213/ANE.0b013e3181ee7f4f

350. Skipper GE, Campbell MD, Dupont RL. Anesthesiologists with substance use disorders: a 5-year outcome study from 16 state physician health programs. *Anesthesia and analgesia*. 2009;109(3):891-6. doi:https://dx.doi.org/10.1213/ane.0b013e3181adc39d

351. Skipper GE, DuPont RL. The Physician Health Program: A replicable model of sustained recovery management. *Addiction recovery management: Theory, research and practice*. 2011;(Anonymous. (1973). The sick physician. Impairment by psychiatric disorders, including alcoholism and drug dependence. JAMA. 1973;223(6):684-7.Crawshaw, R. (1980). An epidemic of suicide among physicians on probation. JAMA. 1980;243 (19):1915-17.Dennis, M.):281-299.

352. Skipper GE, Fletcher C, Rocha-Judd R, Brase D. Tramadol Abuse and Dependence Among Physicians. *JAMA: Journal of the American Medical Association*. 2004;292(15):1818-1819. doi:https://dx.doi.org/10.1001/jama.292.15.1818-b

353. Smith BE. Does an impaired clinician threaten your practice? Legal considerations. *MGMA connexion*. 2004;4(6):5-6.

354. Snashall D. Doctors with disabilities: Licensed to practise? *Clinical Medicine, Journal of the Royal College of Physicians of London*. 2009;9(4):315-319. doi:http://dx.doi.org/10.7861/clinmedicine.9-4-315

355. Soman SG, Jayaprakash P, Joseph JT. How sane are we in this insane world? the role of wellness programs in the identification and management of Impaired Physician. *Indian Journal of Psychiatry*. 2020;62(7 Supplement 1):S121-S122.

356. Soonsawat A, Ahmed I, Lammando M. How does cognitive aging affect clinical competence of physicians? Current status of age-based mandatory cognitive testing of physicians. *American Journal of Geriatric Psychiatry*. 2017;25(3 Supplement 1):S16-S17.

357. Soonsawat A, Ellison J, Ahmed I. Physician with cognitive impairment. *American Journal of Geriatric Psychiatry*. 2015;23(3 SUPPL. 1):S131-S132.

358. Soonsawat A, Tanaka G, Lammando MA, Ahmed I, Ellison JM. Cognitively Impaired Physicians: How Do We Detect Them? How Do We Assist Them? *The American journal of geriatric psychiatry : official journal of the American Association for Geriatric Psychiatry*. 2018;26(6):631-640. doi:https://dx.doi.org/10.1016/j.jagp.2018.01.203

359. SoRelle R. Older surgeons' records for endarterectomy worse than those of younger surgeons. *Circulation*. 2000;102(20):E9040.

360. Spickard A, Swiggart WH, Manley G, Dodd D. A continuing education course for physicians who cross sexual boundaries. *Sexual Addiction & Compulsivity: The Journal of Treatment & Prevention*. 2002;9(1):33-42. doi:https://dx.doi.org/10.1080/107201602317346629

361. Spickard WA, Jr., Swiggart WH, Manley GT, Samenow CP, Dodd DT. A continuing medical education approach to improve sexual boundaries of physicians. *Bulletin of the Menninger Clinic*. 2008;72(1):38-53. doi:https://dx.doi.org/10.1521/bumc.2008.72.1.38

362. Srivastava AB, Gold MS. Impaired physicians. *The Cambridge handbook of substance and behavioral addictions*. 2020;(Alexander, B. H., Checkoway, H., Nagahama, S. I., & Domino, K. B. (2000). Cause-specific mortality risks of anesthesiologists. Anesthesiology, 93(4), 922-930.American Psychiatric Association. (2000). Diagnostic and Statistical Manual of Mental Disorders:):326-332. doi:https://dx.doi.org/10.1017/9781108632591.033

363. Stacy JH. Impaired physicians and the MMPI-2: Comparison of profiles by impairment type. *Dissertation Abstracts International: Section B: The Sciences and Engineering*. 2018;78(10-B(E)):No-Specified.

364. Stergiopoulos E, Martimianakis MAT, Zaheer J. Questioning physicians about health conditions at medical licensure registration: How should policy evolve in Canada? *CMAJ : Canadian Medical Association journal = journal de l'Association medicale canadienne*. 2023;195(20):E710-E716. doi:https://dx.doi.org/10.1503/cmaj.221097

365. Stolbach A, Nelson LS, Hoffman RS. Protection of patients from physician substance misuse. *JAMA: Journal of the American Medical Association*. 2013;310(13):1402-1403. doi:https://dx.doi.org/10.1001/jama.2013.277948

366. Struckmann V, Panteli D, Legido-Quigley H, Risso-Gill I, McKee M, Busse R. Deciding when physicians are unfit to practise: an analysis of responsibilities, policy and practice in 11 European Union member states. *Clinical medicine (London, England)*. 2015;15(4):319-24. doi:https://dx.doi.org/10.7861/clinmedicine.15-4-319

367. Sudan R, Seymour K. The Impaired Surgeon. *The Surgical clinics of North America*. 2016;96(1):89-93. doi:https://dx.doi.org/10.1016/j.suc.2015.09.006

368. Summer GL. Physician impairment: current concepts. *Alabama medicine : journal of the Medical Association of the State of Alabama*. 1994;64(4):24-5.

369. Tanksley AL, Wolfson RK, Arora VM. Changing the "Working While Sick" Culture: Promoting Fitness for Duty in Health Care. *JAMA*. 2016;315(6):603-4. doi:https://dx.doi.org/10.1001/jama.2016.0094

370. Taub S, Morin K, Goldrich MS, Ray P, Benjamin R. Physician health and wellness. *Occupational medicine (Oxford, England)*. 2006;56(2):77-82.

371. Tawfeeq S, Singh A, Dilip Mehta P, et al. SLEEP DISORDERS AMONG PHYSICIANS: A SYSTEMATIC REVIEW OF PREVALENCE, IMPACT ON BURNOUT, AND PATIENT SAFETY. *Chest*. 2023;164(4 Supplement):A6314. doi:https://dx.doi.org/10.1016/j.chest.2023.07.4067

372. The L. Recognising physicians with impairment. *Lancet (London, England)*. 2019;393(10189):2360. doi:https://dx.doi.org/10.1016/S0140-6736(19)31356-X

373. Tischler V. Dr Junkie. The Doctor Addict in Bulgakov's Morphine: What are the Lessons for Contemporary Medical Practice? *The Journal of medical humanities*. 2015;36(4):359-68. doi:https://dx.doi.org/10.1007/s10912-013-9259-z

374. Trockel MT, Menon NK, Rowe SG, et al. Assessment of Physician Sleep and Wellness, Burnout, and Clinically Significant Medical Errors. *JAMA network open*. 2020;3(12):e2028111. doi:https://dx.doi.org/10.1001/jamanetworkopen.2020.28111

375. Tsugawa Y, Newhouse JP, Zaslavsky AM, Blumenthal DM, Jena AB. Physician age and outcomes in elderly patients in hospital in the US: observational study. *BMJ (Clinical research ed)*. 2017;357(8900488, bmj, 101090866):j1797. doi:https://dx.doi.org/10.1136/bmj.j1797

376. Turnbull J, Carbotte R, Hanna E, et al. Cognitive difficulty in physicians. *Academic medicine : journal of the Association of American Medical Colleges*. 2000;75(2):177-81.

377. Turnbull J, Cunnington J, Unsal A, Norman G, Ferguson B. Competence and cognitive difficulty in physicians: a follow-up study. *Academic medicine : journal of the Association of American Medical Colleges*. 2006;81(10):915-8.

378. Tyssen R. Health problems and the use of health services among physicians: a review article with particular emphasis on Norwegian studies. *Industrial health*. 2007;45(5):599-610.

379. van Bogaert DK, Ogunbanjo GA. Ethics in health care: "Physician, heal thyself". *South African Family Practice*. 2014;56(1):S14-S16.

380. Vayr F, Herin F, Jullian B, Soulat JM, Franchitto N. Barriers to seeking help for physicians with substance use disorder: A review. *Drug and alcohol dependence*. 2019;199(ebs, 7513587):116-121. doi:https://dx.doi.org/10.1016/j.drugalcdep.2019.04.004

381. Veerapen RJ. Informed consent: physician inexperience is a material risk for patients. *The Journal of law, medicine & ethics : a journal of the American Society of Law, Medicine & Ethics*. 2007;35(3):478-85.

382. Voelker R. Finding effective treatment for impaired physicians. *JAMA*. 1994;272(16):1238.

383. Waljee JF, Greenfield LJ. Aging and surgeon performance. *Advances in surgery*. 2007;41(2pj, 0045335):189-98.

384. Walker YN. Protecting the public: The impact of the Americans with Disabilities Act on licensure considerations involving mentally impaired medical and legal professionals. *Journal of Legal Medicine*. 2004;25(4):441-468. doi:https://dx.doi.org/10.1080/01947640490887562

385. Warhaft NJ. The Victorian Doctors Health Program: the first 3 years. *The Medical journal of Australia*. 2004;181(7):376-9.

386. Waters PM, Williams D. Later in Career Surgeon Performance Assessment: Why, When, What, and by Whom. *Journal of Pediatric Orthopaedics*. 2021;41(5):322-326. doi:https://dx.doi.org/10.1097/BPO.0000000000001795

387. Weenink J-W, Kool RB, Bartels RH, Westert GP. Getting back on track: a systematic review of the outcomes of remediation and rehabilitation programmes for healthcare professionals with performance concerns. *BMJ quality & safety*. 2017;26(12):1004-1014. doi:https://dx.doi.org/10.1136/bmjqs-2017-006710

388. Weinhouse S, Merlo LJ, Bundy CC, et al. Barriers to recovery for medical professionals: Assessing financial support through a survey of Physician Health Programs. *The American journal on addictions*. 2023;32(4):385-392. doi:https://dx.doi.org/10.1111/ajad.13397

389. Welle D, Trockel MT, Hamidi MS, et al. Association of Occupational Distress and Sleep-Related Impairment in Physicians With Unsolicited Patient Complaints. *Mayo Clinic proceedings*. 2020;95(4):719-726. doi:https://dx.doi.org/10.1016/j.mayocp.2019.09.025

390. West CP, Dyrbye LN, Shanafelt TD. Physician burnout: contributors, consequences and solutions. *Journal of internal medicine*. 2018;283(6):516-529. doi:https://dx.doi.org/10.1111/joim.12752

391. Wettstein RM. Commentary: Quality improvement and psychiatric fitness-for-duty evaluations of physicians. *The journal of the American Academy of Psychiatry and the Law*. 2005;33(1):92-4.

392. Wijeratne C. Professional capacity and the late career medical practitioner. *International Psychogeriatrics*. 2015;27(SUPPL. 1):S185-S186. doi:https://dx.doi.org/10.1017/S1041610215002173

393. Wile C, Frei M, Jenkins K. Doctors and medical students case managed by an Australian Doctors Health Program: characteristics and outcomes. *Australasian psychiatry : bulletin of Royal Australian and New Zealand College of Psychiatrists*. 2011;19(3):202-5. doi:https://dx.doi.org/10.3109/10398562.2011.561846

394. Wilhelm KA, Lapsley H. Disruptive doctors. Unprofessional interpersonal behaviour in doctors. *The Medical journal of Australia*. 2000;173(7):384-6.

395. Wilhelm KA, Reid AM. Critical decision points in the management of impaired doctors: the New South Wales Medical Board program. *The Medical journal of Australia*. 2004;181(7):372-5.

396. Williams BW. The prevalence and special educational requirements of dyscompetent physicians. *The Journal of continuing education in the health professions*. 2006;26(3):173-91.

397. Williams BW, Flanders P. Physician health and wellbeing provide challenges to patient safety and outcome quality across the careerspan. *Australasian psychiatry : bulletin of Royal Australian and New Zealand College of Psychiatrists*. 2016;24(2):144-7. doi:https://dx.doi.org/10.1177/1039856215626652

398. Williams BW, Flanders P, Welindt D, Williams MV. Importance of neuropsychological screening in physicians referred for performance concerns. *PLoS ONE*. 2018;13(11)doi:https://dx.doi.org/10.1371/journal.pone.0207874

399. Winter RO, Birnberg B. Working with impaired residents: trials, tribulations, and successes. *Family medicine*. 2002;34(3):190-6.

400. Wold P, Karlin S. Psychiatric issues in physician impairment. *Rhode Island medicine*. 1994;77(10):351-3.

401. Xing-mao Z, Hui-rong H, Zi-nian W, et al. Age, is it an obstacle for older surgeons to learn laparoscopic approach for colorectal cancer? *Medical oncology (Northwood, London, England)*. 2013;30(2):495. doi:https://dx.doi.org/10.1007/s12032-013-0495-x

402. Yancey JR, McKinnon HD, Jr. Reaching out to an impaired physician. *Family practice management*. 2010;17(1):27-31.

403. Yellowlees PM, Campbell MD, Rose JS, et al. Psychiatrists with substance use disorders: positive treatment outcomes from physician health programs. *Psychiatric services (Washington, DC)*. 2014;65(12):1492-5. doi:https://dx.doi.org/10.1176/appi.ps.201300472
