# Supplementary material for: Health conditions that impact fitness-to-practice in physicians: a scoping review
Source: Int J Qual Health Care. 2025 Oct 7;37(4):mzaf108. doi: 10.1093/intqhc/mzaf108 (PMC12596707; doi:10.1093/intqhc/mzaf108)
Supplement: mzaf108_Supplementary_Data [file mzaf108_supplementary_data.zip › Supplemental File 7.docx]

| **Addiction (not further specified)** | Addiction (where not further specified) |
| --- | --- |
| **Aging and age-related issues** | Aging, younger age |
| **Dexterity / fine motor skills / psychomotor performance** | Dexterity / fine motor skills / psychomotor performance |
| **Hearing** | Hearing |
| **Vision** | Vision |
| **Alcohol use and related conditions** | Diagnoses: alcohol use disorder, Korsakoff's syndrome Alcohol use: alcohol use/abuse/misuse/dependence/addiction/drinking problem/alcoholism |
| **Drug/substance use and related conditions** | Diagnoses: cannabis use disorder, hallucinogen use disorder, narcotics use disorder, opioid use disorder, polydrug abuse, sedative/anxiolytic/hypnotic use disorder, stimulant use disorder, tobacco use disorder, substance use/abuse/misuse/dependence/addiction, substance use disorders Drug use: drug side effects, alpha-II inhibitors, amphetamines, analgesics, antidepressants, anti-seizure medications, anxiolytics, atypical antipsychotics, benzodiazepines, beta-blockers, buprenorphine, cathinones (khat), chloroform, coffee/caffeine, crack cocaine, cocaine, depressants, diphenhydramine, ether, fentanyl, gamma hydroxybutyrate (GHB) / gamma butyrolactone (GBL), hallucinogens, heroin, inhalants, ketamine, lysergic acid diethylamide (LSD), marijuana/cannabis, 3,4-methylenedioxymethamphetamine (MDMA, ecstasy), methadone, methamphetamine, mood stabilizers, muscle relaxants, mushrooms, narcotics, nitrous oxide, opioid agonists, opiates, opioid agonist/substitution therapy, phencyclidine (PCP), performance-enhancing medications, pethidine/Demerol, piperazines, propofol, psychotropic medications, sedative-hypnotics, steroids, stimulants, synthetic cannabinoids, tobacco/nicotine use, tramadol, tranquilizers |
| **Mental health issues** | Acute stress disorder, attention-deficit/hyperactivity disorder (ADHD), adjustment disorders, affective disorders, antisocial personality disorder, anxiety/anxiety disorders, autism spectrum disorder, Axis I disorder, behavioural addictions, behavioural disorders, bereavement, bipolar disorder, borderline personality disorder, burnout, Cluster B personality disorder, compulsive behaviour, compulsive gambling, cyclothymia, delusional disorder, dependent personality disorder, depression, dissociative disorder, dyslexia, dysthymic disorder, eating disorders, hypomania, impulse control disorder, intermittent explosive disorder, kleptomania, learning disability, major depressive disorder, malingering, mania, mental health/mental illness, mood disorder, narcissistic personality disorder, obsessive-compulsive disorder, paranoia, paranoid personality disorder, paraphilias, personality disorder/Axis II disorder, personality disorder not otherwise specified (NOS), process addiction, psychiatric disorders/illness, psychosexual disorders, psychosis/psychotic disorders, post-traumatic stress disorder (PTSD), pyromania, schizoaffective disorder, schizophrenia, seasonal affective disorder, specific phobia, speech impairment, stress, suicidality |
| **Neurological conditions** | Alzheimer's disease, brain disease, cerebral palsy, stroke, cerebrovascular disease, cognitive/cognition, cognitive disorders, delirium, dementia, epilepsy, headaches, memory, mild cognitive impairment (MCI), movement disorders, multiple sclerosis (MS), neurological disorder, organic personality change, paresthesia, Parkinson's disease, processing speed/reaction time, sensory/perceptual, seizure disorder, traumatic brain injury (TBI), transient ischemic attack (TIA), transient global amnesia |
| **Medical conditions (other than neurological, hearing, vision)** | Acute infectious or viral diseases, anemia, arthritis, cancer, cardiac, cataracts, chronic infectious diseases, chronic fatigue syndrome/myalgic encephalomyelitis (ME), chronic pain, chronic respiratory diseases, circadian rhythm disruption, clubfoot, coronary artery disease, dehydration, dermatological disorders, diabetes, endocrine disorders, fatigue/endurance, genetic abnormalities, gastrointestinal (GI) disorders, glaucoma, hepatitis, hepatitis (HBV) infection, hepatitis C, hepatic cirrhosis, hepatic disorders, hepatic encephalopathy, high blood pressure, human immunodeficiency virus (HIV)-positive/HIV-infected, hyperlipidemia, hypotension, immunosuppression, influenza-like illness, inflammatory bowel disease, injury, malnutrition/vitamin deficiency, medication withdrawal compensation, metabolic disorder, musculoskeletal, osteoporosis/osteopenia, physical capacity/function, physical disability/impairment, physical illness, renal disease/disorder, reproductive disorders, sleep apnea, sleep deprivation impairments, somatic symptoms, syphilis, tuberculosis, ulcerative colitis, vascular disease |
| Legend: **Each separate individual category is indicated by a *comma* delimiter.** | |
